# CO organization at ambient pressure on stepped Pt surfaces: first principles modeling accelerated by neural networks†

**DOI:** 10.1039/d1sc03827c

**Published:** 2021-11-15

**Authors:** Vaidish Sumaria, Philippe Sautet

**Affiliations:** Department of Chemical and Biomolecular Engineering, University of California Los Angeles CA 90094 USA sautet@ucla.edu; Department of Chemistry and Biochemistry, University of California Los Angeles CA 90094 USA

## Abstract

Step and kink sites at Pt surfaces have crucial importance in catalysis. We employ a high dimensional neural network potential (HDNNP) trained using first-principles calculations to determine the adsorption structure of CO under ambient conditions (*T* = 300 K and *P* = 1 atm) on these surfaces. To thoroughly explore the potential energy surface (PES), we use a modified basin hopping method. We utilize the explored PES to identify the adsorbate structures and show that under the considered conditions several low free energy structures exist. Under the considered temperature and pressure conditions, the step edge (or kink) is totally occupied by on-top CO molecules. We show that the step structure and the structure of CO molecules on the step dictate the arrangement of CO molecules on the lower terrace. On surfaces with (111) steps, like Pt(553), CO forms quasi-hexagonal structures on the terrace with the top site preferred, with on average two top site CO for one multiply bonded CO, while in contrast surfaces with (100) steps, like Pt(557), present a majority of multiply bonded CO on their terrace. Short terraced surfaces, like Pt(643), with square (100) steps that are broken by kink sites constrain the CO arrangement parallel to the step edge. Overall, this effort provides detailed analysis on the influence of the step edge structure, kink sites, and terrace width on the organization of CO molecules on non-reconstructed stepped surfaces, yielding initial structures for understanding restructuring events driven by CO at high coverages and ambient pressure.

## Abstract 2

A neural network potential trained using first-principles calculations enables to understand the adsorption configurations of carbon monoxide on stepped Pt surfaces at ambient pressure.

## Graphical abstract

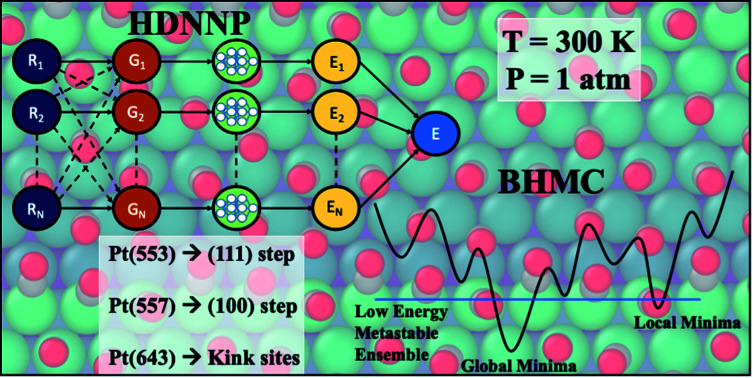

## 1 Introduction

The active phase of transition metal heterogeneous catalysts presents atoms in different coordinations and environments. Surface science experiments over the last two decades have systematically studied the relationship between the surface structure and catalytic activity by using single-crystal surfaces as model catalysts. Open surface structures or surfaces with a high Miller index often show enhanced activity.^1–5^ The high-index planes, denoted by a set of Miller indices (*hkl*) with at least one index being larger than one, have a high density of atomic steps and kinks. The low coordination atoms which define the atomic step/kink sites on the catalyst surface often enable enhanced binding of reactant molecules and exhibit higher activity for bond breaking.^6–13^ At the same time, steps also play an important role in other surface processes like adsorbate induced reconstructions. The surface atoms at the step/kink site act as natural locations for crystal growth and erosion and as a source for mobile surface ad-atoms during the process of surface reconstruction.^14–19^ This makes it important to study the assembly structure of adsorbate molecules on such stepped surfaces under realistic temperature and pressure conditions (which defines the adsorbate chemical potential) to understand the adsorption site distribution and the adsorbate coverage. Such a high coverage adsorbate structure is the initial configuration for adsorbate induced surface reconstruction processes at such stepped and kink surface sites. In this work, we focus our attention on the Pt/CO system to understand the CO organization on Pt(553), Pt(557) and Pt(643) surfaces at room temperature and ambient pressure. All these surfaces show (111) terraces, separated by (111) and (100) mono-atomic steps for Pt(553) and Pt(557) respectively. Pt(643) is a case where the step includes kinks, hence presenting Pt atoms with a metallic coordination of 6.

Since the considered step surfaces all present (111) terraces, the behavior and organization of CO on the extended Pt(111) surface are key references. CO adopts on Pt(111) multiple ordered structures depending on the coverage. At 300 K and 1 atm pressure of CO, experiments (such as scanning tunneling microscopy) and first principles modelling show that CO molecules adopt a so called “Moiré pattern” structure, in which CO, binding vertically through the C atom, is organized in a pseudo-hexagonal layer with a rotated supercell with respect to the underlying 1 × 1 hexagonal Pt layer. More specifically a 
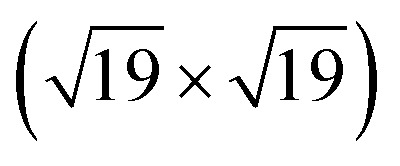
–*R*23.4°–13 CO unit cell is found where 13 CO molecules occupy a cell containing 19 Pt atoms, corresponding to a coverage of 13/19 = 0.68 ML (see ESI S4†).^20–23^ Since the CO quasi-hexagonal layer has a slightly larger parameter than the Pt(111) layer, CO molecules span a variety of binding sites, from (quasi) top to bridge and hollow. Although the classification between these various sites relies on chosen thresholds and hence is somewhat ambiguous, one can determine that, among the 13 CO molecules, 7 are in a top or quasi top site, while 4 are in bridge/quasi-bridge and 2 in hollow sites. So about one half of CO molecules are on the top site, while the other half is in a multiply bonded site. Many experiments are performed under UHV conditions with a small CO pressure and low temperature to reach a high CO coverage. Under these conditions, the CO coverage is typically somewhat lower (0.5 ML) and a simple *c*(4 × 2)–2CO superstructure is seen, with one top and one bridge CO. Hence, a very similar ratio (0.5) is seen between top and multiply bonded CO, compared to the high pressure Moiré pattern arrangement.

If we now move to stepped and kinked surfaces, the experimental characterization of adsorbate layer surfaces is challenging. These surfaces are heterogeneous, with different types of sites (step, kink, and terrace), and adsorption is usually less ordered. For example, Tränkenschuh *et al.* did not find any LEED pattern for CO adsorption on Pt(553), showing an absence of long range order, in contrast to Pt(111).^24^ This absence of long range order was also seen on many other high index surfaces (Pt(533),^25^ Pt(332),^26^ Pt(210)^27^ and Pt(321).^28^ Spectroscopic characterization methods necessarily give space average information. For CO on stepped Pt surfaces, high resolution X-ray photoelectron spectroscopy (XPS) and electron energy loss spectroscopy (EELS) can distinguish between CO adsorbed at step sites and terrace sites, and between top and bridge bonded CO at each site.^15,24,26,29–31^ On Pt(553), XPS at low pressure (less than 3 × 10^−9^ mbar) and low temperature (130 K) shows that the terrace is only occupied when the edge sites are almost saturated with CO, while on the terrace the population of the top sites is about twice that of the bridge sites at a saturation coverage of ∼0.5 ML.^24^ This contrasts with the case of Pt(111) where at a similar coverage the population of top sites equals that of bridge sites. On Pt(335) at 0.5 ML coverage, EELS also shows that all edge sites are occupied with top CO, while on the terrace half of the sites are occupied with a 2 : 1 top-to-bridge ratio.^26^

*In situ* FTIR studies under solution and electrochemical conditions indicate that the preferred binding mode of CO on the terrace of stepped surfaces with short terraces depends on the type of step: surfaces with (100) steps (Pt(322) and Pt(311)) show a large amount of bridge site CO on their terraces, while those with (111) steps (Pt(332) and Pt(331)) mostly provide top site CO.^32^ This suggests an interesting mechanism, of unknown origin to our knowledge, by which the type of step can control the binding site of CO on the terraces. Near-field microscopies, such as scanning tunneling microscopy, provide images with molecular resolution, but the detailed interpretation of the images can be challenging, and time resolution is usually limited. STM images of 0.5 ML of CO on a Pt(111) surface presenting steps show less order and higher mobility of CO molecules in the vicinity of the step.^33^

Computational studies that investigate high Miller index surfaces with a high adsorbate coverage are very limited. The complexity of modeling such surfaces arises from two major challenges: (1) the high computational cost of exploring such a potential energy surface (PES) using accurate first principles calculations and (2) the myriad combinations of adsorption sites possible at high coverages. To tackle the first challenge, we use the approach developed by Behler and Parrinello to generate a High Dimensional Neural Network Potential (HDNNP)^34^ that is trained on higher-level first principles calculations and can accurately describe the adsorbate–adsorbate and adsorbate–surface interactions on various high Miller surfaces. This approach is ideal for studying these systems because it scales favorably with respect to the dimensionality of the system and guarantees the permutation invariance of the PES due to the conversion of unique local environments into unique fingerprints. In the past few years, NN based methods have been increasingly used to construct such an accurate PES successfully.^35–43^ To tackle the second challenge of exploring the large combination of adsorption sites possible, we use a Basin-hopping algorithm.^44^ This method transforms the PES into local minima basins and the transition between these basins is accomplished by performing a Monte–Carlo displacement trial move, followed by geometry optimization. Such a method is not very often applied while using DFT to compute the free energy of the system to evaluate the MC criteria due to the high cost of DFT calculations and due to the fact that a significantly large number of MC steps can be required to reach a global minimum. Having an accurate and quick energy calculator in terms of the HDNNP, we can efficiently generate a large number of configurations of CO on different Pt surfaces for a given coverage and chemical potential of CO.

In most theoretical studies, the PES is not thoroughly sampled and only a few local minima are found, the most stable one being supposed to represent the structure of the adsorbates on the substrate. This is due to the unaffordable cost of modeling and exploring the PES as we discussed before. However, it is now known that the pressure of the adsorbates can generate dynamic interfaces which can play a nontrivial role in understanding the actual distribution of the adsorbates on the surface. The majority of the discussion of such dynamic states in the literature has been focused on understanding the fluxionality of small nanoparticles and nanostructured surfaces.^45–50^ Sun *et al.* discussed the case of a Pt_13_ cluster under a pressure of hydrogen, found a large ensemble of low energy structures, and showed that these structures change with hydrogen coverage. Metastable structures are shown to dominate the catalytic activity. One observation made in the case of Pt_13_H_26_ structures was that all the structures in the metastable ensemble exhibited a cuboctahedral Pt_13_ core, and the only variation in the structures was produced by the hydrogen atoms occupying different positions on the cluster. This indicates that metastable structures can be generated by just rearrangement of adsorbate atoms and hence that the concept of metastable ensembles can also be applied to a gas–surface interaction. In this work, using basin hopping simulations, we not only search for the global minima for CO adsorption structures on various stepped surfaces, but also find the coverage dependent ensemble of possible CO configurations. We show that the CO organization on the terrace is strongly affected by the geometry of the step ((100) or (111) facets), which controls the ratio between top and multiply bonded CO molecules, that quasi-hexagonal CO lattices are formed on these terraces similarly to the case of Pt(111)^17,21^ and that step edges are in most cases fully covered with one top site CO molecule on each step Pt atom.

## 2 Results and discussion

### 2.1 Pt(553)

**Fig. 1:**
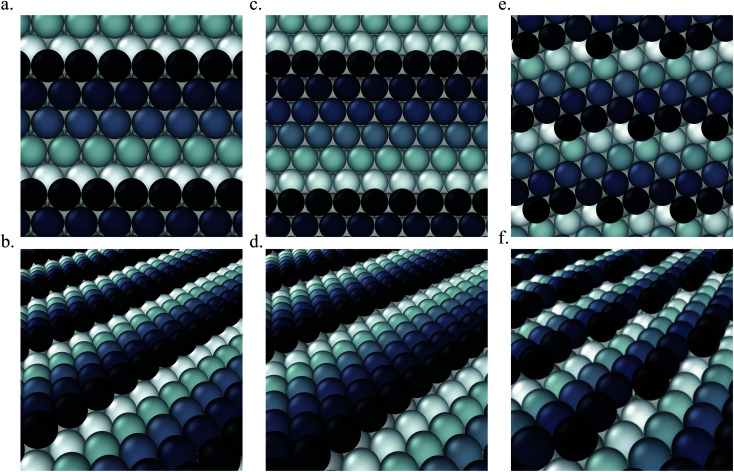
Ball models of various clean surfaces considered. (a) Pt(553) top view, (b) Pt(553) side view, (c) Pt(557) top view, (d) Pt(557) side view, (e) Pt(643) top view, and (f) Pt(643) side view. Colors represent the *z*-position of the atoms. Black balls represent the step edge and others represent the terrace.

Pt(553) can be represented as Pt(S)-[5(111) × (111)] in step notation since it is formed by a 5 atom wide Pt(111) terrace followed by a mono-atomic 111-type step as shown in Fig. 1(a) and (b). One challenge is that no long-range order for CO is found experimentally on such stepped surfaces, while a unit cell is required to model the surface. We tackle this by considering several unit cells, that describe a rather large area of the step and terrace. This is possible only because we obtain a fast and accurate neural network potential enabling the sampling of a very large number of configurations for CO molecules, at variable coverage, on these unit cells. Such a sampling would be extremely computationally costly using DFT calculations. For Pt(553) we use 3 × 1, 4 × 1, and 6 × 1 unit cells, with the first periodic direction being along the step edge while the second one (x1) corresponds to the (long) periodicity between one step and the next one. With these unit cells, we can explore the various organisation periodicities of molecules along the step edge using basin hopping simulations.

**Fig. 2:**
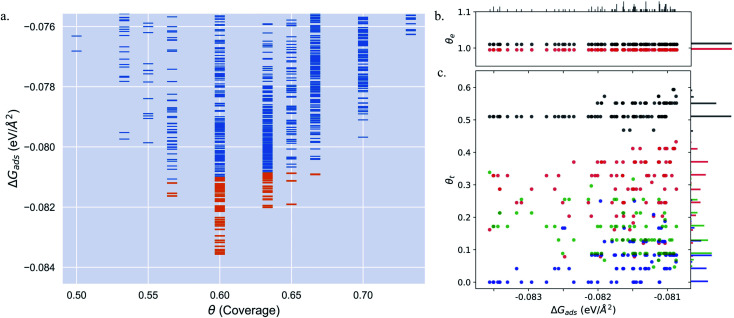
(a) Adsorption free energy per unit surface area Δ*G*_ads_ (at *T* = 300 K and *P* = 1 atm) plotted against CO coverage (*θ*) on Pt(553). Yellow markers represent the structures defending the low energy minima ensemble. (b and c) The coverage of CO on the Pt step-edge and terrace as a function of Δ*G*_ads_ respectively. 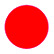 represents the top site, 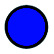 represents the bridge site, 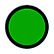 represents the hollow site and ● represents the total coverage. The density of different sites at various coverages is represented by the histogram attached to the right ordinate axis. The density of structures as a function of Δ*G*_ads_ is represented by the histogram on the top abscissa axis. In plots (b) and (c), to distinguish the points, we move the red points (top sites) on the *y*-axis by −0.005, green points (hollow sites) have been moved by +0.005 and black points (total coverage) have been moved by +0.01.

We calculate the total Gibbs free energy of adsorption for all CO molecules, including entropy terms for the gas phase molecules but neglecting the vibrational entropy for adsorbed molecules. This adsorption energy is then normalized to a 1 Å^2^ surface area, to be able to compare different unit cells. Fig. 2(a) shows the Gibbs adsorption free energy of CO per unit surface area on Pt(553) as a function of CO coverage. A total number of 1501 local minima configurations have been explored, and their free energy is shown by dashed lines in Fig. 2(a), with the orange lines (97 structures) representing the low energy accessible region, called here the low energy metastable ensemble (LEME). The energy interval for the LEME is chosen to be 1 kT per CO adsorbate. The LEME found on the Pt(553) surface consists of structures with a coverage between *θ* = 0.55 and *θ* = 0.67. So the first clear comment is that we do not find a single stable structure, and CO coverage for the considered *T* and *P* conditions, but in contrast, a large number of competing low energy structures are seen corresponding to a range of coverage. This simply explains the absence of long range order in the experiment.

Common points and differences between these 97 structures in the LEME can be seen in Fig. 2, where the coverage for various CO binding modes (top, bridge, and hollow) is indicated on the step edge (Fig. 2(b)) and on the terrace (Fig. 2(c)), as a function of the adsorption free energy per unit surface area. The data used to create this plot have been included in the ESI (see Table S1†). All structures in the LEME have the step edge completely populated by on-top CO molecules, with one CO per Pt atom. The coverage of the terrace ranges between 0.5 and 0.6 ML, where on average on the LEME structures, top site occupation is dominating (average coverage 0.3 ML), followed by hollow sites (average coverage 0.14) and bridge sites (average coverage 0.07). Therefore, the number of top CO molecules is on average about twice that of the multiply bound CO on the terrace of Pt(553).

**Fig. 3:**
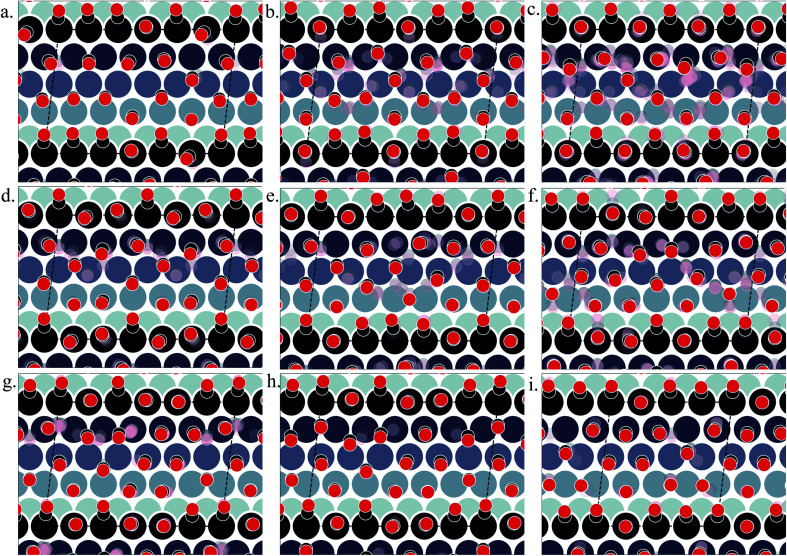
Various structures of CO orientation patterns observed in the LEME for Pt(553). (a) Structures with *θ* = 0.56; (b)–(e) structures with *θ* = 0.6; (f)–(h) with *θ* = 0.63; (i) with *θ* = 0.65. The Pt atoms are shown using a colormap (color-bar represents the *z*-position of the Pt atom). For a given pattern, the lowest energy orientation of CO is shown using the solid black and red balls representing the positions of C and O respectively and the ensemble of structures is represented by the “cloud” of pink and grey balls.

Fig. 3(a)–(i) show the structural arrangement of CO at various coverages between 0.55 and 0.67 found in the LEME. For a given most stable structural arrangement of CO on the Pt(553) surface at a given coverage, small variations in the CO positions generate structures that have adsorption free energy within the energy window. From the LEME configurations obtained using Basin Hopping Monte Carlo (BHMC) simulations, unique configurations are identified by converting the chemical environment into graph representations using the SurfGraph code developed by Deshpande *et al.*^51^ The most energetically favourable structure (lowest Gibbs free energy) is shown by the red and black balls in Fig. 3(a)–(i) and the small variations are shown using the “clouds” of pink and grey balls (representing the oxygen and carbon atoms respectively).

As proposed by the experiments, the step edge is totally covered by on-top CO molecules.^24,26,32^ CO adsorption results from the competition between stabilizing CO-surface interactions and destabilizing CO–CO lateral interactions that increase with CO coverage. Hence, the CO equilibrium coverage depends on the strength of the molecule–surface interaction and on the CO chemical potential (governed by *T* and *P*). Step sites correspond to a lower Pt coordination and hence a stronger CO–Pt interaction than the (111) terraces (see ESI Section 4). At the step, a coverage of 1 CO per Pt can be reached, but CO molecules present a different angular orientation to limit the CO–CO interactions. Such CO orientation leads to tilt angles from −25° to 46°, where negative angles represent a tilt towards the upper terrace and positive angles represent a tilt towards the lower terrace. Different tilt motifs are seen along the step edge with x2, x3, or x6 periodicities providing quasi-degenerate energies. At a chemical potential of −15.29 eV (corresponding to *T* = 300 K and *P* = 1 atm), the most favorable coverage of CO is found to be 0.6 ML (as seen in Fig. 2(a)), with 54 structures in the LEME with this coverage. Fig. 3(b)–(e) represent the four CO arrangement patterns observed at this coverage (in decreasing stability order). In these structures, we find that CO molecules on the terrace arrange in distorted hexagonal patterns, the denser atomic directions being tilted ∼25–36° with respect to the Pt step edge. These arrangements are limited by the short width of the terraces, but mimic the Moiré-like patterns seen on Pt(111). CO on the step Pt atoms interestingly arranges in a manner such that the quasi-hexagonal pattern on the lower terrace can be maintained. In a way the modulation of the CO tilt angle along the step initiates the organisation of molecules on the lower terrace. Descending from the step to the lower terrace, the highly coordinated Pt atoms at the bottom of the step are not populated. The lack of CO at the bottom of the step is also observed at other coverages throughout the LEME structures for Pt(553) and can be attributed to reducing the repulsion between the on-top step CO and the CO on the lower terrace. In contrast, the Pt row beyond the step edge on the upper terrace shows a high occupation of CO (0.5–0.83 ML). The coverage of 1 ML at the step edge implies a smaller coverage on the terrace of 0.5 ML. The C–C distance at the densely populated step is between 2.8 and 3.0 Å (compared to 2.818 Å for the Pt–Pt distance), whereas the first C–C neighbor distance on the terrace varies between 3.2 and 3.4 Å, underlining the lower CO density on the terrace coming from a weaker adsorbate–surface interaction.

Three structures in the LEME are found at *θ* = 0.56 and are shown in Fig. 3(a). At this low coverage, we don't see any systematic arrangement of CO on the terrace, but the step edge still has a 1 ML CO coverage. At *θ* = 0.63, we see 3 different patterns (33 structures) of CO arrangement on Pt(553) shown in Fig. 3(f)–(h) with increasing adsorption free energy order. All the structures at this coverage have a 6 × 1 periodicity with a tilt angle of −17° to 44° along the fully populated step edge. The CO coverage on the terrace is 0.54 (13 CO atoms on 24 Pt atoms). The CO arrangement on the terrace is less ordered compared to the arrangement found at *θ* = 0.6, making the quasi-hexagonal pattern less visible. At *θ* = 0.65, we observe only a 4 × 1 unit cell periodicity along the step edge and 4 structures manifest in the LEME structures (Fig. 2(i)). Similar to the structures found at *θ* = 0.6, we find that CO molecules on the terrace arrange in distorted hexagonal patterns with the denser atomic directions being tilted ∼55° with respect to the Pt step edge. A higher mobility of CO molecules is observed near the upper terrace, while the CO positions near the lower terrace and step edge remain approximately constant among the 4 structures. The C–C distance on the step with all CO molecules on the top site is between 2.8 and 3.0 Å and the first C–C neighbor distance on the terrace varies between 3.0 and 3.2 Å. At *θ* = 0.67, we find one structure in the LEME with a 6 × 1 periodicity and CO arranging along parallel lines that are angled 55° (anti-clock wise) with respect to the step edge (see ESI Fig. S5†).

Overall, for Pt(553), the low energy metastable ensemble of structures has a coverage between 0.56 and 0.65. The short terrace length in combination with the (111)-type step only allows clear quasi-hexagonal arrangements of CO. The low coordination Pt atoms at the step site and the possibility of varying tilt angles of CO around the step edge enable a higher local coverage with CO adsorbing on the top site. On the terrace, the C–C first neighbor distance decreases as the coverage increases to incorporate more CO on the surface. Comparing the site distribution on the terrace, we see that the majority of the structures have a higher top site coverage followed by hollow site occupation and the least occupied sites are the bridge sites.

### 2.2 Pt(557)

**Fig. 4:**
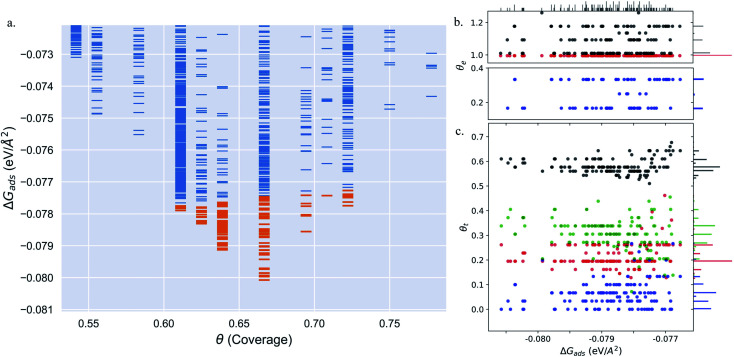
(a) Adsorption free energy per unit area (Δ*G*_ads_) plotted against CO coverage (*θ*) on Pt(557). Yellow markers represent the structures defining the LEME. (b and c) The coverage of CO on the Pt step-edge and terrace as a function of Δ*G*_ads_ respectively. 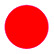 represents the top site, 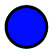 represents the bridge site, 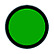 represents the hollow site and ● represents the total coverage. The points in (b) and (c) have been moved in a similar fashion to Fig. 2 to distinguish the points better.

**Fig. 5:**
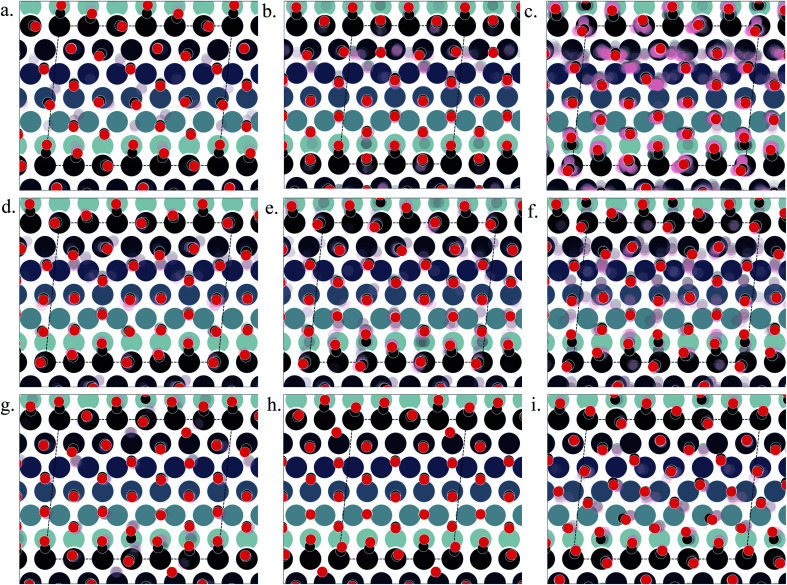
Various structures of CO orientation patterns observed in the LEME for Pt(557). (a) Structures with *θ* = 0.61, (b) with *θ* = 0.625, (c) with *θ* = 0.64, (d–f) with *θ* = 0.66, (g) with *θ* = 0.69, (h) with *θ* = 0.71, and (i) with *θ* = 0.72. The Pt atoms are shown using a colormap (color-bar represents the *z*-position of the Pt atom). For a given pattern, the lowest energy orientation of CO is shown using the solid black and red balls representing the positions of C and O respectively and the ensemble of structures is represented by the “cloud” of pink and grey balls.

Pt(557) can be represented as Pt[6(111) × (100)] in step notation since it is formed by a 6 atom wide Pt(111) terrace followed by a mono-atomic 100-type step as shown in Fig. 1(c) and (d). We use 3 × 1, 4 × 1, and 6 × 1 unit cells to explore the CO organization on the surface. The low energy CO configurations on Pt(557) at RT and 1 atm CO pressure found by our HDNNP and basin hopping approach correspond to coverages between 0.61 ML and 0.72 ML with 142 structures in the LEME (Fig. 4(a)). Similar to Pt(553), the step site is completely occupied by on-top CO molecules. However, the more open character of the (100) steps permits to have in addition CO molecules bridging between a step edge atom and one Pt immediately below in the lower terrace. The step edge atom can hence be bound to two CO molecules, one top and one bridge. In that case (as seen in Fig. 5(c, e–g and i)), the top molecule is not leaning towards the lower terrace, but towards the upper terrace. With that mechanism, the coverage at the step can reach up to 1.25 ML (the bridging CO molecule is shared between the two Pt atoms (Fig. 4(b))). The uneven tilt of the CO molecules at the step edge initiates the organisation on the terrace, with quasi-hexagonal ordering throughout the LEME and helps incorporate higher coverage on the square step. The tilt angles range between −20° and 50° with x2 and x3 periodic motifs that provide quasi-degenerate energies. For the structures in the LEME, on the terrace, the coverage ranges from 0.50 to 0.67 ML, with on average, hollow sites dominating with a coverage of 0.3 ML, top sites with 0.22 ML and bridge sites at 0.06 ML (Fig. 4(c)). Hence, on average, on the terrace, multiply bounded CO on the terrace is on average almost twice that of the top site. This strongly contrasts with the previous case of Pt(553), where the on-top CO was dominating on the terrace, indicating that the type of step has a strong influence on the configuration of CO molecules.

Under 300 K and 1 atm conditions, the most stable structure is found at *θ* = 0.67. At this coverage, we find three types of CO arrangements as shown in Fig. 5(d)–(f) (in the stability order). For the most stable structure at *θ* = 0.67, CO arranges along parallel lines that are tilted ∼30° with respect to the Pt step edge (Fig. 5(d) – arrangement shown using black and red balls). Such a positioning of CO molecules on the terrace, which is accompanied by the CO molecules creating an x3 motif on the step with varying tilts towards the lower terrace, helps create a quasi-hexagonal pattern. The change of CO positions at the lower terrace (mainly between hollow and bridge sites) results in quasi-degenerate LEME structures that have been represented by the pink and gray “clouds” in Fig. 5. At the same coverage, we find another orientation of CO at the step edge (with an x2 motif) with alternating CO molecules tilting towards and away from the lower terrace (Fig. 5(e)). At this coverage, the C–C distance (first neighbor) varies between 2.7 and 3.18 Å on the terrace and 2.8–3.18 Å on the step edge.

Within the LEME, lower coverage structures are found with *θ* = 0.61 (4 structures), *θ* = 0.625 (12 structures), and *θ* = 0.64 (59 structures). At higher coverages, the LEME includes structures with *θ* = 0.69 (6 structures), *θ* = 0.71 (1 structure), *θ* = 0.72 (8 structure). At these coverages, the quasi-hexagonal pattern is maintained on the terrace and the step coverage remains 1 ML as seen in Fig. 5. The CO quasi-hexagonal lattice rotation with respect to the step edge is coverage dependent. At *θ* = 0.61, CO arranges along parallel lines that are tilted ∼23.5°. At *θ* = 0.625 and *θ* = 0.64, the angle increases to 30° and at *θ* = 0.66 the tilt angle further increases to ∼40°. Further increase in coverage leads to reduction in the tilt angle to 30–35° at *θ* = 0.69 and *θ* = 0.71 and to ∼20° at *θ* = 0.72. The different rotation angles of the CO lattice allow maintaining approximately similar C–C first neighbor distances on the terrace (∼3.15–3.22), while allowing more CO to be incorporated on the surface. Though out the LEME ensemble, the step manifests an x2 or x3 repeating motif. Except at *θ* = 0.61 and *θ* = 0.71, all the other structures in the LEME show additional CO molecules bridging between the step edge and lower terrace allowing for incorporating more CO on the surface. This increases the step edge coverage to 1.17 ML.

Overall, for Pt(557), the low energy metastable ensemble of structures has a coverage between 0.61 and 0.72, slightly higher than the case of Pt(553). The formation of a more ordered hexagonal pattern for the CO adsorbates on the Pt substrate can be attributed to the longer terrace length and the square Pt(100) step. One striking difference concerns the binding sites of CO on the terrace, with the dominant multiply bonded CO on the (557) surface, while top sites were twice more numerous on the (553) termination. The more open Pt(100) step allows CO bridging between the terrace and the step edge which in turn allows the hexagonal pattern of the CO lattice to continue across the stepped surface. These three factors have a significant impact on the CO arrangement on the surface.

### 2.3 Pt(643)

**Fig. 6:**
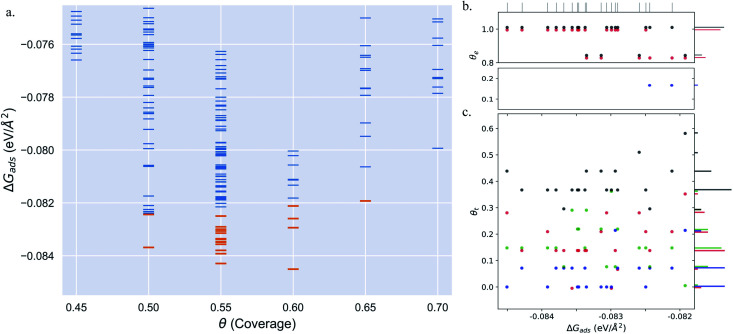
(a) Adsorption free energy per unit area (Δ*G*_ads_) plotted against CO coverage (*θ*) on Pt(643). Yellow markers represent the structures defining the LEME. (b and c) The coverage of CO on the Pt step-edge and terrace as a function of Δ*G*_ads_ respectively. 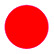 represents the top site, 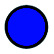 represents the bridge site, 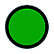 represents the hollow site and ● represents the total coverage. The points in (b) and (c) have been moved in a similar fashion to Fig. 2 to distinguish the points better.

The Pt(643) surface consists of Pt(111) terraces separating Pt(100) steps that are broken by Pt(110) kink sites. The kink shows a convex site on a 6-coordinated Pt atom, next to a concave site on an 8-coordinated Pt atom (Fig. 1(e) and (f)). Basin hopping simulations using the neural network potential were run on a (2 × 1) unit cell (creating a 6 Pt atom step edge with the kink site) which showed a CO coverage from 0.5 to 0.65 ML (Fig. 6(a)) in the low energy minima ensemble of structures (20 structures) under 300 K and 1 atm conditions, with the most stable structure having a coverage of 0.6 ML. The coverage of CO on the Pt step edge (*θ*_e_) is shown in Fig. 6(b). In the ensemble, 4 structures show *θ*_e_ = 0.83 ML and 16 structures show *θ*_e_ = 1.0 ML. Two structures (with *θ*_e_ = 1 ML and *θ* = 0.7 ML) have CO on the bridge site at the concave site of the kink, which are shown using blue points on Fig. 6(b). For all the other structures, CO occupies the top site on the step edge and kink atoms. Compared to Pt(553) and Pt(557), Pt(643) has a smaller terrace length which does not allow a similar rotated hexagonal pattern arrangement of CO. In contrast, the short terrace combined with the kink site and the square step edge forces the CO organization parallel to the dense Pt atom arrangement throughout the LEME structures. The short terrace also limits the number of structures in the LEME, the adsorbate layer being less fluxional.

**Fig. 7:**
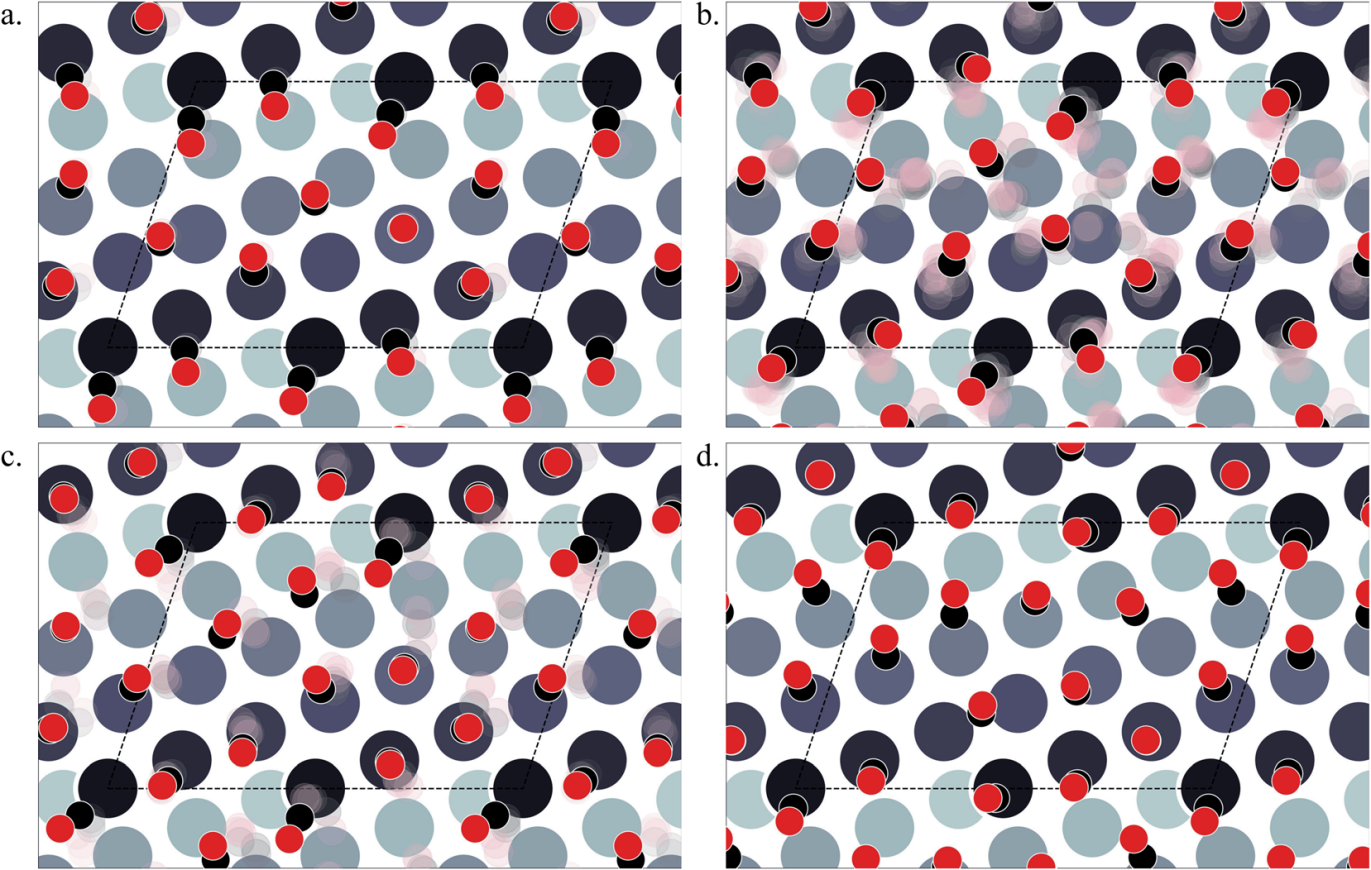
Various structures of CO orientation patterns observed in the LEME for Pt(643). (a) Structures with *θ* = 0.5; (b) with *θ* = 0.55; (c) with *θ* = 0.6; (d) with *θ* = 0.65. The Pt atoms are shown using a colormap (color-bar represents the *z*-position of the Pt atom). For a given pattern, the lowest energy orientation of CO is shown using the solid black and red balls representing the positions of C and O respectively and the ensemble of structures is represented by the “cloud” of pink and grey balls.

At *θ* = 0.5, 2 structures exist in the LEME (Fig. 7(a)). At this coverage, the step sites are fully covered with on-top CO and the terrace top to bridge ratio is 1 : 1. The CO assembly at this coverage is less ordered, which results in more distributed positions. At *θ* = 0.55, 13 structures ((Fig. 7(b))) are observed in the LEME, all of which except two structures with low coordination step Pt atoms have a *θ*_e_ = 1 ML and the remaining two have a *θ*_e_ = 0.83 ML. At this coverage, on average over the LEME structures, the top to hollow site occupation ratio on the terrace is approximately 1 : 1, while bridge site occupation is negligible. The tilt of CO molecules at the kink atom can be large (∼40°). On the terrace, at *θ* = 0.55, the parallel CO arrangement is broken near the kink site.

The most stable structure on Pt(643) is found at a *θ* = 0.6 and is shown in Fig. 7(c). Similar to the structures at *θ* = 0.55, CO aligns along lines that are parallel to the step edge but the symmetry is maintained throughout. In the ensemble at this coverage, *θ*_e_ varies from 0.83–1 ML and all CO molecules adsorbed at the step occupy the top site. CO molecules on the terrace occupy the top (on average 0.23 ML) and hollow sites (on average 0.125 ML) with only minor (or no) occupation of bridge sites. The short terrace and parallel arrangement of CO with respect to the substrate Pt atoms possibly do not allow the bridge site occupation. At *θ* = 0.65, we find only 1 structure in the LEME as shown in Fig. 7(d) where we observe similar arrangement of CO as for *θ* = 0.55, where the CO alignment pattern is broken near the kink Pt atom site. At this coverage, once again top sites dominate the occupation on the terrace with 0.36 ML.

Overall, on Pt(643), we see a combined effect of a kink site, a square step and a small terrace length which leads to an arrangement of CO that aligns parallel to the step edge. On average across the LEME structures we find an equal occupation of top and hollow sites while bridge occupation is limited. Such a behaviour is unique compared to Pt(553) and Pt(557) where we saw a development of hexagonal patterns of CO arrangement. Understanding the CO organization on such a surface with kinks and steps can be important since the low coordination kink site appearing on this surface can show specific catalytic activity and also can be the starting point of the restructuring event under CO pressure.

## 3 Conclusion

The organization of CO adsorbates on different surfaces including steps and kinks and presenting (111) terraces was modelled under an ambient pressure of CO at room temperature, by combining a neural network potential, trained using first principles structures, including energies and forces, and a basin hopping approach to efficiently explore the various configurations of the CO adsorbates, describing an extensive number of structures (∼4500). The main conclusions are summarized below. Under the considered conditions, the step edge (or kink) is totally occupied by on-top CO molecules, while terraces show a partial occupation close to 0.5 ML. One recurring feature is that the surface (Pt(553), Pt(557) or Pt(643)) does not show a single most stable CO adsorption structure at the considered CO chemical potential, but that a large number of adsorbate configurations on the terrace are close in energy. By considering a threshold of 1 kT per CO adsorbate, we defined the LEME, ensemble of structures thermally accessible, which contain 97, 142 and 20 distinct structures for the considered Pt(553), Pt(557) and Pt(643) unit cells respectively. These structures show imperfect quasi-hexagonal ordering on the terraces, while the arrangement along the step edge is more ordered, including different tilts with respect to the terrace normal to decrease CO–CO interactions. The large number of competing structures should result in a mixture of very small domains of different configurations, and hence an absence of long range order, as seen in the experiments. Some common results arise from this study. The first one concerns the distribution between top and multiply bonded CO on the (111) terraces. Stable adsorption configurations on the Pt(553) surface present more top site CO than multiply bonded ones on the (111) terrace, resulting on average in two top site CO for one multiply bonded CO. The situation is completely modified on the (111) terraces of the (557) surface, where in on average two multiply bonded CO are seen for one top CO. On the kinked Pt(643) surface, the top CO molecules again dominate on the surface. These results should be compared with the extended Pt(111) surface where under the same conditions an equal number of top and multiply bonded CO molecules are found. The three considered surfaces show different structures of the step edge: a (111) step for Pt(533), a (100) step for Pt(557) and a kink site with a short terrace for Pt(643).

Since the binding energy of top CO is very close to that of multiply bonded CO on the (111) terrace, the structure of the step plays a key role in initiating the arrangement of CO on the lower terrace. The CO at the step with their different angles of tilt towards the terrace acts as a boundary condition to organize the adsorbates on the terrace.

The (111) steps favor the construction of quasi-hexagonal layers where top site CO dominates, presenting alignments of CO at various possible angles with respect to the step edge, while in contrast the (100) steps pin configurations with a large fraction of multiply bonded sites.

Our calculated results agree well with the experimental data from Farias *et al.* under electrochemical conditions where Pt(322) and Pt(311) surfaces, that present a (100) step, show a large amount of bridge site CO on their terraces, while Pt(332) and Pt(331) surfaces, with a (111) step, show a very large majority of top site CO.^32^ They also agree with the UHV experiments of Tränkenschuh *et al.* in the case of Pt(553)).^24^ The kink surface (Pt(643)) shows a different type of arrangement where CO aligns parallel to the step edge. This demonstrates that the atomic arrangement at the steps controls the structure of the CO ad-layers at least in a zone close to the step edge.

Understanding CO adsorption at stepped surfaces is a prerequisite to study their catalytic reactivity in reactions involving CO. It is also of key importance as the initial structure leading to surface restructuring upon CO adsorption at ambient pressure. Indeed, extended Pt step edges are known to undergo reconstruction driven by CO adsorption at high coverage. Our study opens the way to realistic modelling of these restructuring events.

## 4 Methods

### 4.1 First principles calculation details

Calculations were performed using the Vienna *Ab initio* Simulation Package^52–55^ using the general gradient approximation (GGA) Perdew–Burke–Ernzerhof (PBE) functional.^56^ Core electrons were described using projector augmented wave potentials.^57,58^ A *k*-spacing of 0.25 is used for all the calculations and the generated *k*-point grid is centered at the *Γ* point. Periodic slabs of the Pt surface with CO are separated by a 12 Å vacuum in the *z* direction. A Fermi smearing width of 0.2 eV was applied using the Methfessel–Paxton method (order 2). A cutoff energy of 400 eV is used. The known issue of overbinding of CO on Pt surfaces (“Pt(111)/CO Puzzle”) has been corrected using the CO bond distance-based correction developed by us.^20^ The generalized correction is given as *Δ* = 4.77 × *d*_CO_ − 5.37, where *Δ* is the correction applied in (eV) and *d*_CO_ is the bond length of adsorbed CO in (Å).

### 4.2 Training a high dimensional neural network potential

We exploit the use of a HDNNP to define the atomic potential for a Pt/CO system to reduce the computational cost and explore the potential energy surface (PES) efficiently. The assumption in developing the HDNNP is that there exists a unique functional relationship between the atomic coordinates and the potential energy. Using the HDNNP, the total energy of the system is defined as the sum of individual atomic contributions, *E*_s_ = ∑*E*_*i*_, where the atomic energy *E*_*i*_ is found by training atomic neural networks using structural and chemical environments (*E*_s_ = ∑*NN*(*X*^env^_*i*_)). These environments are defined using various feature transformations (descriptors) that convert Cartesian coordinates, which are not invariant with respect to the translation and rotation of the system to invariant representations implemented using atom-centered symmetry functions (ACSF),^59^ smooth overlap of atomic positions (SOAP),^60^ many-body tensor representation (MBTR),^61^*etc.*

In this work, we use the high-dimensional neural network potential where the *R*^*α*^_*i*_ (Cartesian coordinates *α* of atom i) are transformed into a set of symmetry function values *G*^*μ*^_*i*_ for each atom *i*.^62^ The developed NN utilises 2 hidden layers with 30 nodes each and a hyperbolic tangent activation function. A total of 46 symmetry functions were used for each element – Pt, C, and O. We use the weighted atom-centered symmetry function (wACSF) proposed by Gastegger *et al.* which can be expressed as:^63^1
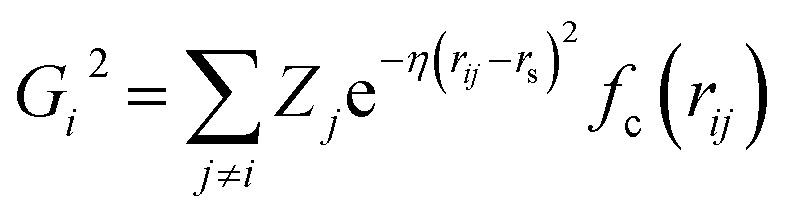
2
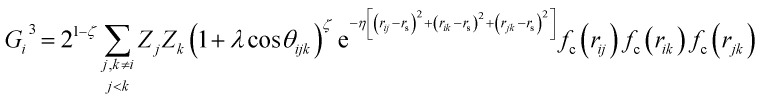

3 *f*_c_(*r*_*ij*_) = ((15 − 6*r*_*ij*_)*r*_*ij*_ − 10)*r*_*ij*_^3^ + 1

where *G*_*i*_^2^ represents the radial symmetry function and *G*_*i*_^3^ represents the angular symmetry function. *f*_c_ represents a polynomial cutoff function. *r*_*ij*_ = |*r*_*i*_ − *r*_*j*_| represents the interatomic distance between atoms *i* and *j* and *θ*_*ijk*_ = (*r*_*ij*_·*r*_*ik*_)/(|*r*_*ij*_||*r*_*ik*_|) represents the angle spanned by the atoms *i*, *j*, and *k*. *Z*_*i*/*j*/*k*_ represents the atomic numbers which are used as weights for the symmetry functions. The parameters *η* and *r*_s_ have been determined using the automatic selection algorithm developed by Imbalzano *et al.* based on equally dividing the cutoff radius (*r*_c_ = 6 Å) in *n* intervals which is chosen here to be 5. Two sets of radial symmetry functions (*G*^2^) are used: (i) the first group centered on the reference atom (*r*_s_ = 0) with *η* = 0.0278, 0.0529, 0.1007, 0.1916, 0.3648, 0.6944 Å^−2^ (6 symmetry functions) and (ii) the second group is centered along the path between the central atom and its neighbour with *r*_s_ = 1.5, 2.1213, 3, 4 Å and *η* = 2.5904, 1.2952, 0.6476, 0.3238 Å^−2^ respectively (4 symmetry functions). 36 angular symmetry functions (*G*^3^) have been defined with *r*_s_ = 0; *η* = 0.0278, 0.0529, 0.1007, 0.1916, 0.3648, 0.6944 Å^−2^; *λ* = −1,1 and *ζ* = 1, 4, 16. This creates atomic NN architectures that can be represented as 46–30–30–1.

The reference database is built iteratively. Starting from an initial reference data set (consisting of a Pt(111) surface with different unit cell sizes and CO coverages), a first preliminary HDNNP is obtained. This HDNNP is used with BHMC simulations to generate more relevant data, which are used to validate the potential to check for extrapolations and missing Potential Energy Surface (PES) data. If the accuracy of the developed potential is insufficient, problematic structures are identified and added to the training set until a converged potential is obtained. Fig. S3 in the ESI† shows a flowchart explaining this iterative process of HDNNP development. In every iteration of the training, 10% of the total reference data generated are used as a validation fraction and over fitting is avoided by using the early stop algorithm. The final reference database consisted of 4289 structures. We used the n2p2 package for training.^64^ This package provides an efficient approach for optimizing the weight parameters of the neural network *via* multi-stream Kalman filtering, using potential energies and atomic forces as reference data. A loss function for the training is defined as the sum of energy and force RMSE (root mean square error), *i.e.*, force coefficient *γ* = 1. For the re-usability of the developed potential, we include the output weight parameter and other files in the ESI.† The “input.nn” file includes all variables needed to reproduce the NN as well, including symmetry functions, the cutoff radius, and hidden layers and nodes and the “weights.*.data” contains the weights of the atomic NNs.

### 4.3 Basin hopping Monte Carlo (BHMC) simulation

The basin hopping method is a frequently used global optimization method for finding low energy structures. For our purposes, we use the method to generate new structures by taking advantage of the efficient HDNNP to quickly sample the configuration space that can be then recalculated by DFT to add to the reference database. This algorithm takes advantage of the basin hopping method and Monte Carlo method with local minimization to convert the potential energy surface (PES) from a curved surface to stepped shape basins.^65^ The exploration of these basins is achieved by Monte Carlo sampling through atomic displacements and the Metropolis criterion. The free energy is calculated by subtracting the reference chemical potential (which is a function of temperature and pressure) of the adsorbate from the energy of the system as shown by the following equation:

4 Δ*G* = *E*(*n*CO + slab) − *E*(slab) − *n*_CO_ × *μ*_CO_

where *E*(*n*CO + slab) is the electronic energy of the optimized structure, *E*(slab) is the energy of the optimized bare Pt slab and *μ*_CO_ is the chemical potential of CO in the gas phase. Translational and rotational terms are taken into account to calculate the CO gas phase chemical potential, but vibrational terms are not included, since they are neglected in the CO adsorbed state as well.

**Fig. 8:**
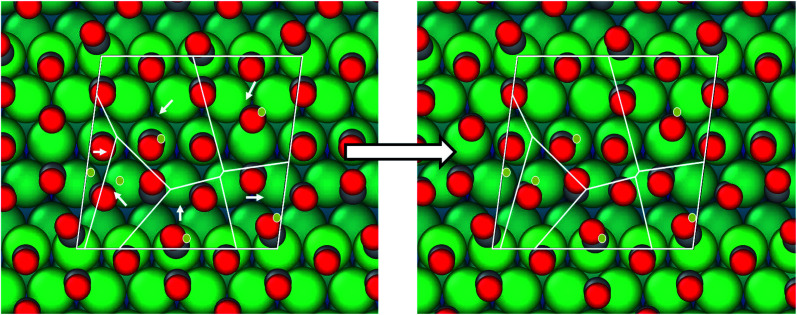
Clustering mutation algorithm implemented with the basin hopping algorithm. The dark gray balls represent carbon atoms and red balls represent oxygen atoms. The light gray balls are the substrate platinum atoms. The blue lines define the Voronoi tessellation and the yellow dots represent the centers for the Voronoi clusters. Atoms within one cluster are moved randomly in the same direction as seen in the model implementation.

The Metropolis criterion implies that a MC move is always accepted if the free energy of the new structure Δ*G*_new_ is lower than the previous structure, Δ*G*_old_; otherwise it is accepted with a probability   exp[(Δ*G*_old_ − Δ*G*_new_)/*k*_B_*T*_MC_], which is determined by a random number drawn from the interval [0, 1]. Here, the “temperature” for BH simulation (*T*_MC_) is an adjustable parameter. Based on the acceptance and rejection of the structures while running the BH simulation, this parameter can be adjusted. A flow chart explaining the BHMC algorithm used in this work has been included in the ESI (Fig. S1 and S2†). The BHMC algorithm differs from the standard MC algorithm in one step namely the local optimization that is performed at each point of the PES and since the BHMC exploration is performed by hopping among different basins, a larger atomic displacement (Δ*r*_*i*_) can be used compared to standard MC. Both these features of BHMC simulations help increase the success rate in obtaining the global minima. Most BHMC implementations utilize random atomic displacements.^66–68^ For high coverage of CO on Pt surfaces, random perturbation of adsorbate atoms leads to a low acceptance ratio (∼20%) since the motion of one CO molecule on the surface is only allowed if the neighboring adsorbed molecules move in a concerted manner. As a result, we introduced a new kind of move, “Clustering mutation algorithm”, where we cluster a few adsorbed molecules randomly and move all in the same random motion together. This was done by dividing the surface into Voronoi tessellations using random center points within the unit cell (Fig. 8). Such a move in conjunction with random atomic displacements significantly improved the exploration of the potential energy surface and increased the acceptance ratio (∼50%). The algorithm is explained in more detail in the ESI.†

A HDNNP is efficiently used with BHMC to explore the PES and find the low energy minima ensemble of structures. Since the HDNNP is not fully accurate, the ordering of structures based on energies is not the same as predicted by the HDDNP when compared with DFT calculations. As a result, we recalculate with DFT all structures that have free energy *G* ≤ *G*_min_ + 0.05 eV Å^−2^ which is a significantly higher error margin than the error expected from the HDNNP. This ensures that we have recalculated all the low energy minima ensemble of structures, which is then used to report the relative adsorption free energies and the minima structures. The (i) BHMC code, (ii) training, validation and test dataset and (iii) HDNNP files are made available on Github.^69^

## Data availability

Data has been made available at GitHub at https://github.com/vsumaria/Pt_CO_steps_NNP.

## Author contributions

V. S. performed DFT calculations, HDNNP training and data analysis for the work. P. S. supervised the project and both the authors discussed the results and helped in writing the manuscript.

## Conflicts of interest

There are no conflicts to declare.

## Supplementary Material

SC-012-D1SC03827C-s001
