# Supplementary material for: CO organization at ambient pressure on stepped Pt surfaces: first principles modeling accelerated by neural networks
Source: Chem Sci. 2021 Nov 15;12(47):15543–55. doi: 10.1039/d1sc03827c (PMC8654054; doi:10.1039/d1sc03827c)
Supplement: SC-012-D1SC03827C-s001 [file SC-012-D1SC03827C-s001.pdf]

Electronic Supplementary Material (ESI) for Chemical Science.
This journal is © The Royal Society of Chemistry 2021

Supporting Information for "CO organization at ambient pressure on stepped Pt surfaces: First principle modeling accelerated by neural networks"

Vaidish Sumaria[†] and Philippe Sautet[*,†,‡]

†Department of Chemical and Biomolecular Engineering, University of California, Los Angeles, CA 90094, USA

‡Department of Chemistry and Biochemistry, University of California, Los Angeles, CA 90094, USA

E-mail: sautet@ucla.edu

1 Basin Hopping Algorithm

Fig. S1 shows the Basin hopping algorithm utilized in the work to generate structures efficiently. Structure updates are done using trail moves in two ways: (1) Rattling (random atomic displacements) the CO molecules to change CO positions and (2) Clustering mutation algorithm.

1.1 Clustering mutation algorithm

The following steps are used in implementing this modified version of random atomic displacement.

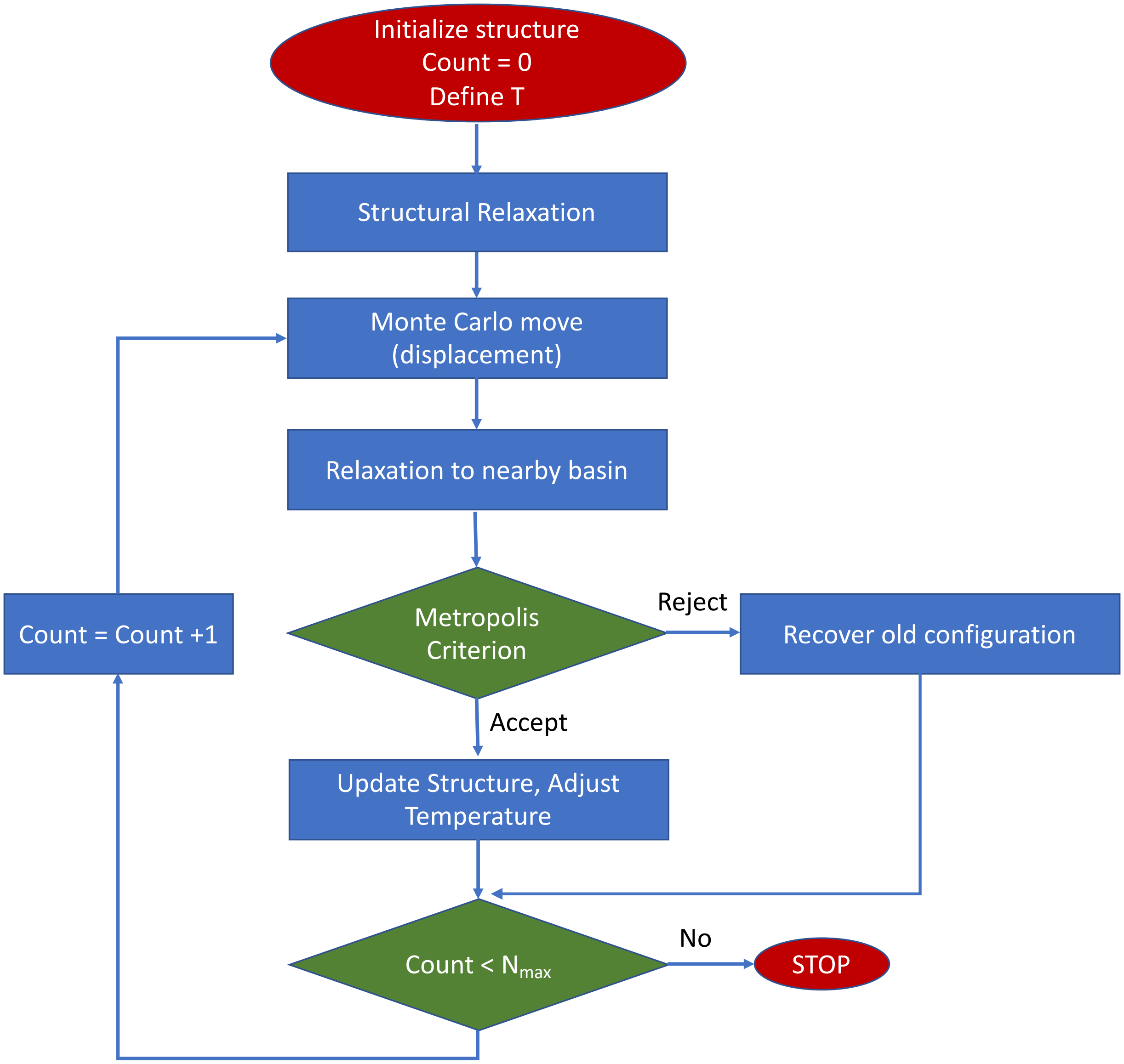

Figure S1: Basin Hopping Algorithm flowchart.

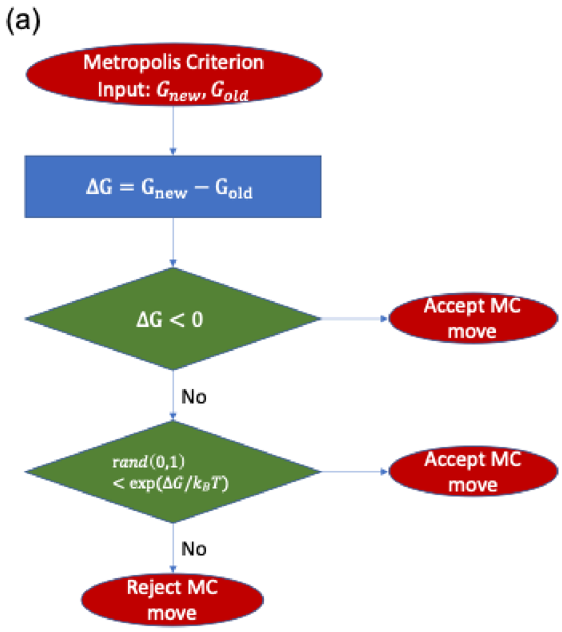

Figure S2: Flow-chart showing the algorithm for (a) Metropolis criterion - which decides the acceptance/ rejection of MC move and (b) adjusting temperature during the BH simulation where (a) is the acceptance ratio for the BH simulation.

1. Create a polygon (parallelogram) that maps the surface of the unit cell

2. Randomly generate n points within the polygon which act as the centroids of the Voronoi tessellation (like a power diagram). Using the polygon boundary and centroids, we can define the edges of the Voronoi tessellation (intersections of half-spaces).

3. Within the each obtained cell ("cluster"), we identify the adsorbate positions and all CO molecules within the cell are displaced in same direction randomly generated.

This is implemented using a pythonic code (rand_clustering.py) using scipy and shapely packages and added to the github repository.

1.2 HDNNP Training procedure

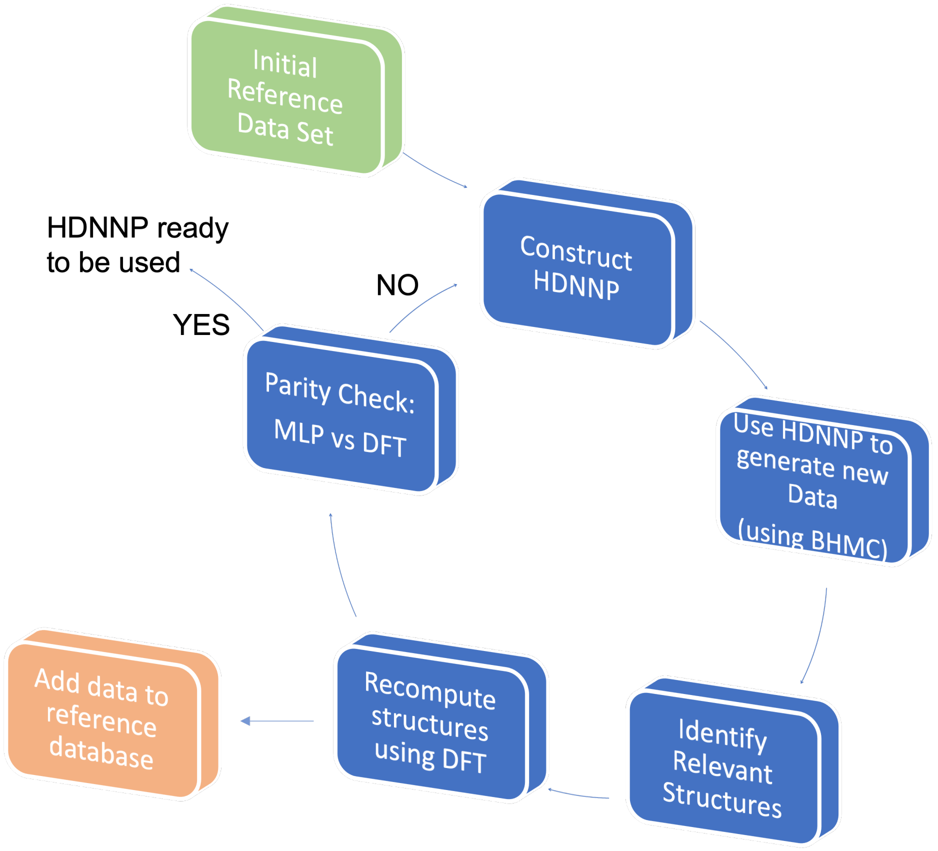

Figure S3: Iterative process for training NN as well as generating the reference database

We use an iterative process (as shown in in Fig. S3) for developing the HDNNP:

- **Initialization** - reference dataset utilized to generate a preliminary HDNNP.
- **Data Generation**- new structures generated using the developed HDNNP and Basin Hopping Monte Carlo simulations.
- **Re-evaluation** - identify structures that are relevant for evaluation and perform reference DFT calculations on these structures.
- **Parity check** - Compare energies and forces obtained from reference DFT calculations and the NN predictions.

- **Retrain & convergence** - Structures deviating in energy and force predictions with respect to the reference DFT calculations are added back to the training dataset to generate a new iteration of HDNNP.

The initial dataset was generated on a Pt(111) surface with varying CO coverages and unit cell sizes.

2 Pt(111) terrace

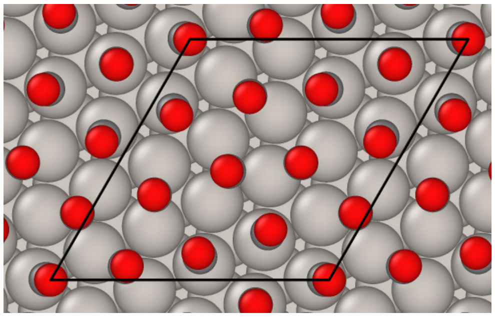

Figure S4: Arrangement of CO on Pt(111) at 300K and 1 atm showing $(\sqrt{19} \times \sqrt{19})$-$R23.4°$-13 CO structure corresponding to a coverage of $13/19 = 0.68$ ML

3 LEME structures data

3.1 Pt(553)

Data used to generate Figure 2 in the manuscript.

Table S1: Pt(553) LEME structures data: Free energy per unit area (G/A), Coverage of CO on the terrace top site (θ_t(T)), bridge site (θ_t(B)), hollow site (θ_t(H)) and on the step edge top site (θ_e(T)), bridge site (θ_e(B)), hollow site (θ_e(H)), total coverage of CO on the terrace (θ_t) and on the step edge (θ_e) and the surface area of the unit cell (A)

G/A (eV/Å^2)	θ_t(T)	θ_t(B)	θ_t(H)	θ_e(T)	θ_e(B)	θ_e(H)	θ_t	θ_e	A(Å^2)
-0.0836	0.17	0	0.33	1	0	0	0.5	1	182.97

-0.0835	0.33	0	0.17	1	0	0	0.5	1	182.97
-0.0835	0.33	0	0.17	1	0	0	0.5	1	182.97
-0.0834	0.33	0	0.17	1	0	0	0.5	1	182.97
-0.0834	0.29	0	0.21	1	0	0	0.5	1	182.97
-0.0834	0.29	0	0.21	1	0	0	0.5	1	182.97
-0.0833	0.33	0	0.17	1	0	0	0.5	1	182.97
-0.0832	0.25	0.04	0.21	1	0	0	0.5	1	182.97
-0.0831	0.25	0	0.25	1	0	0	0.5	1	182.97
-0.083	0.29	0	0.21	1	0	0	0.5	1	182.97
-0.0827	0.33	0	0.17	1	0	0	0.5	1	182.97
-0.0826	0.29	0.04	0.17	1	0	0	0.5	1	182.97
-0.0826	0.33	0.04	0.13	1	0	0	0.5	1	182.97
-0.0825	0.25	0.17	0.08	1	0	0	0.5	1	182.97
-0.0825	0.08	0.17	0.25	1	0	0	0.5	1	182.97
-0.0824	0.21	0.04	0.25	1	0	0	0.5	1	182.97
-0.0823	0.38	0	0.13	1	0	0	0.5	1	182.97
-0.0821	0.25	0	0.25	1	0	0	0.5	1	182.97
-0.0821	0.21	0	0.29	1	0	0	0.5	1	182.97
-0.0821	0.33	0.08	0.08	1	0	0	0.5	1	182.97
-0.082	0.33	0.08	0.08	1	0	0	0.5	1	182.97
-0.082	0.38	0.08	0.08	1	0	0	0.54	1	182.97
-0.082	0.33	0	0.17	1	0	0	0.5	1	182.97
-0.082	0.08	0.25	0.17	1	0	0	0.5	1	182.97
-0.082	0.38	0.08	0.08	1	0	0	0.54	1	182.97
-0.0819	0.38	0.08	0.08	1	0	0	0.54	1	182.97
-0.0819	0.38	0.13	0.06	1	0	0	0.56	1	121.98

-0.0818	0.33	0	0.17	1	0	0	0.5	1	182.97
-0.0818	0.25	0.13	0.13	1	0	0	0.5	1	182.97
-0.0818	0.17	0.08	0.25	1	0	0	0.5	1	182.97
-0.0818	0.29	0.08	0.17	1	0	0	0.54	1	182.97
-0.0817	0.21	0.04	0.25	1	0	0	0.5	1	182.97
-0.0817	0.38	0	0.17	1	0	0	0.54	1	182.97
-0.0817	0.33	0.04	0.13	1	0	0	0.5	1	182.97
-0.0816	0.33	0.08	0.13	1	0	0	0.54	1	182.97
-0.0816	0.33	0.08	0.13	1	0	0	0.54	1	182.97
-0.0816	0.25	0.04	0.21	1	0	0	0.5	1	182.97
-0.0816	0.38	0.04	0.13	1	0	0	0.54	1	182.97
-0.0816	0.25	0.08	0.17	1	0	0	0.5	1	182.97
-0.0816	0.21	0.04	0.21	1	0	0	0.46	1	182.97
-0.0816	0.25	0.17	0.08	1	0	0	0.5	1	182.97
-0.0816	0.29	0.08	0.13	1	0	0	0.5	1	182.97
-0.0815	0.17	0.13	0.17	1	0	0	0.46	1	182.97
-0.0815	0.29	0.13	0.13	1	0	0	0.54	1	182.97
-0.0815	0.42	0.04	0.08	1	0	0	0.54	1	182.97
-0.0815	0.19	0	0.31	1	0	0	0.5	1	121.98
-0.0815	0.21	0.04	0.25	1	0	0	0.5	1	182.97
-0.0815	0.29	0.13	0.08	1	0	0	0.5	1	182.97
-0.0815	0.29	0.08	0.13	1	0	0	0.5	1	182.97
-0.0815	0.38	0	0.13	1	0	0	0.5	1	121.98
-0.0815	0.08	0.25	0.17	1	0	0	0.5	1	182.97
-0.0815	0.38	0.08	0.08	1	0	0	0.54	1	182.97
-0.0815	0.38	0.08	0.08	1	0	0	0.54	1	182.97

-0.0814	0.29	0	0.21	1	0	0	0.5	1	182.97
-0.0814	0.42	0	0.08	1	0	0	0.5	1	182.97
-0.0814	0.29	0.04	0.21	1	0	0	0.54	1	182.97
-0.0814	0.38	0	0.13	1	0	0	0.5	1	182.97
-0.0813	0.13	0.13	0.25	1	0	0	0.5	1	121.98
-0.0813	0.38	0.04	0.13	1	0	0	0.54	1	182.97
-0.0813	0.38	0.08	0.08	1	0	0	0.54	1	182.97
-0.0813	0.38	0.04	0.13	1	0	0	0.54	1	182.97
-0.0813	0.38	0.04	0.13	1	0	0	0.54	1	182.97
-0.0813	0.38	0.04	0.13	1	0	0	0.54	1	182.97
-0.0813	0.25	0.13	0.17	1	0	0	0.54	1	182.97
-0.0813	0.33	0.08	0.08	1	0	0	0.5	1	182.97
-0.0813	0.33	0.04	0.13	1	0	0	0.5	1	182.97
-0.0812	0.31	0.06	0.13	1	0	0	0.5	1	121.98
-0.0812	0.21	0.21	0.04	1	0	0	0.46	1	182.97
-0.0812	0.33	0.08	0.13	1	0	0	0.54	1	182.97
-0.0811	0.38	0.13	0.06	1	0	0	0.56	1	121.98
-0.0811	0.38	0.13	0.06	1	0	0	0.56	1	121.98
-0.0811	0.25	0.17	0.08	1	0	0	0.5	1	182.97
-0.0811	0.13	0.13	0.25	1	0	0	0.5	1	121.98
-0.0811	0.38	0.08	0.08	1	0	0	0.54	1	182.97
-0.0811	0.42	0	0.13	1	0	0	0.54	1	182.97
-0.0811	0.42	0	0.13	1	0	0	0.54	1	182.97
-0.0811	0.25	0.17	0.08	1	0	0	0.5	1	182.97
-0.0811	0.21	0.13	0.17	1	0	0	0.5	1	182.97
-0.0811	0.33	0.13	0.08	1	0	0	0.54	1	182.97

-0.081	0.33	0.13	0.08	1	0	0	0.54	1	182.97
-0.081	0.25	0.04	0.21	1	0	0	0.5	1	182.97
-0.081	0.29	0.13	0.08	1	0	0	0.5	1	182.97
-0.081	0.13	0.19	0.19	1	0	0	0.5	1	121.98
-0.081	0.42	0.04	0.08	1	0	0	0.54	1	182.97
-0.081	0.25	0.08	0.17	1	0	0	0.5	1	182.97
-0.081	0.33	0.08	0.08	1	0	0	0.5	1	182.97
-0.081	0.38	0.08	0.08	1	0	0	0.54	1	182.97
-0.081	0.42	0.04	0.08	1	0	0	0.54	1	182.97
-0.081	0.38	0.08	0.08	1	0	0	0.54	1	182.97
-0.081	0.38	0.04	0.13	1	0	0	0.54	1	182.97
-0.081	0.38	0.08	0.08	1	0	0	0.54	1	182.97
-0.0809	0.33	0.08	0.13	1	0	0	0.54	1	182.97
-0.0809	0.42	0.08	0.08	1	0	0	0.58	1	182.97
-0.0809	0.42	0.08	0.08	1	0	0	0.58	1	182.97
-0.0809	0.42	0.08	0.04	1	0	0	0.54	1	182.97
-0.0809	0.44	0.06	0.06	1	0	0	0.56	1	121.98
-0.0809	0.29	0.08	0.17	1	0	0	0.54	1	182.97

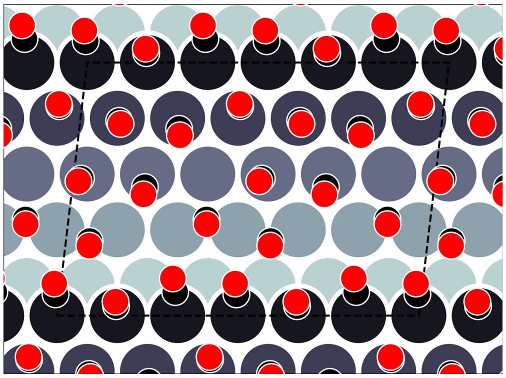

Figure S5: CO orientation on Pt(553) at θ =0.65 in the LEME structure

3.2 Pt(557)

Data used to generate Figure 4 in the manuscript.

Table S2: Pt(557) LEME structures data: Free energy per unit area (G/A), Coverage of CO on the terrace top site (θ_t(T)), bridge site (θ_t(B)), hollow site (θ_t(H)) and on the step edge top site (θ_e(T)), bridge site (θ_e(B)), hollow site (θ_e(H)), total coverage of CO on the terrace (θ_t) and on the step edge (θ_e) and the surface area of the unit cell (A)

G/A (eV/Å^2)	θ_t(T)	θ_t(B)	θ_t(H)	θ_e(T)	θ_e(B)	θ_e(H)	θ_t	θ_e	A(Å^2)
-0.0801	0.27	0	0.33	1	0	0	0.6	1	118.5
-0.08	0.2	0.03	0.37	1	0.17	0	0.58	1.08	237.01
-0.0799	0.2	0	0.4	1	0	0	0.6	1	237.01
-0.0799	0.2	0.1	0.3	1	0.33	0	0.57	1.17	237.01
-0.0799	0.2	0.1	0.3	1	0.33	0	0.57	1.17	237.01
-0.0797	0.2	0.03	0.37	1	0.17	0	0.58	1.08	237.01
-0.0797	0.23	0	0.37	1	0	0	0.6	1	237.01
-0.0797	0.23	0	0.37	1	0	0	0.6	1	237.01
-0.0797	0.23	0.03	0.33	1	0.17	0	0.58	1.08	237.01
-0.0794	0.2	0.2	0.2	1	0.5	0	0.55	1.25	237.01
-0.0793	0.27	0.07	0.27	1	0.33	0	0.57	1.17	118.5
-0.0793	0.17	0.07	0.37	1	0.33	0	0.57	1.17	237.01
-0.0792	0.2	0	0.4	1	0	0	0.6	1	118.5
-0.0792	0.27	0	0.33	1	0	0	0.6	1	237.01
-0.0792	0.2	0	0.4	1	0	0	0.6	1	118.5
-0.0791	0.2	0.07	0.33	1	0.33	0	0.57	1.17	118.5
-0.0791	0.2	0.07	0.33	1	0.33	0	0.57	1.17	118.5
-0.0791	0.2	0.07	0.3	1	0.17	0	0.55	1.08	237.01
-0.0791	0.2	0.03	0.33	1	0	0	0.57	1	237.01
-0.079	0.27	0	0.33	1	0	0	0.6	1	118.5

-0.079	0.27	0.07	0.27	1	0.33	0	0.57	1.17	118.5
-0.079	0.27	0.03	0.27	1	0	0	0.57	1	237.01
-0.079	0.2	0.07	0.3	1	0.17	0	0.55	1.08	237.01
-0.0789	0.2	0.07	0.3	1	0.17	0	0.55	1.08	237.01
-0.0789	0.27	0	0.33	1	0	0	0.6	1	118.5
-0.0789	0.17	0.07	0.33	1	0.17	0	0.55	1.08	237.01
-0.0788	0.2	0.03	0.33	1	0	0	0.57	1	237.01
-0.0788	0.2	0.1	0.27	1	0.33	0	0.53	1.17	237.01
-0.0788	0.2	0.03	0.33	1	0	0	0.57	1	237.01
-0.0787	0.23	0.03	0.3	1	0.17	0	0.55	1.08	237.01
-0.0787	0.2	0.1	0.27	1	0.33	0	0.53	1.17	237.01
-0.0787	0.2	0	0.4	1	0	0	0.6	1	118.5
-0.0787	0.2	0	0.4	1	0	0	0.6	1	118.5
-0.0787	0.27	0.07	0.27	1	0.33	0	0.57	1.17	118.5
-0.0787	0.2	0.07	0.3	1	0.17	0	0.55	1.08	237.01
-0.0787	0.2	0.07	0.33	1	0.33	0	0.57	1.17	118.5
-0.0787	0.2	0.07	0.33	1	0.33	0	0.57	1.17	118.5
-0.0787	0.2	0.03	0.33	1	0	0	0.57	1	237.01
-0.0787	0.17	0.07	0.33	1	0	0	0.57	1	237.01
-0.0786	0.2	0.07	0.3	1	0.17	0	0.55	1.08	237.01
-0.0786	0.27	0.07	0.27	1	0	0	0.6	1	118.5
-0.0786	0.27	0.13	0.2	1	0.33	0	0.57	1.17	118.5
-0.0786	0.27	0	0.33	1	0	0	0.6	1	118.5
-0.0786	0.27	0	0.33	1	0	0	0.6	1	118.5
-0.0786	0.2	0.03	0.33	1	0.17	0	0.55	1.08	237.01
-0.0786	0.2	0	0.43	1	0	0	0.63	1	237.01

-0.0785	0.27	0.07	0.27	1	0	0	0.6	1	118.5
-0.0785	0.23	0	0.33	1	0	0	0.57	1	237.01
-0.0785	0.27	0	0.3	1	0	0	0.57	1	237.01
-0.0785	0.2	0.07	0.33	1	0	0	0.6	1	118.5
-0.0785	0.2	0.07	0.33	1	0	0	0.6	1	118.5
-0.0785	0.2	0.07	0.3	1	0.17	0	0.55	1.08	237.01
-0.0785	0.2	0.07	0.33	1	0.33	0	0.57	1.17	118.5
-0.0785	0.2	0.03	0.33	1	0	0	0.57	1	237.01
-0.0785	0.2	0.07	0.33	1	0.33	0	0.57	1.17	118.5
-0.0785	0.2	0.13	0.27	1	0.33	0	0.57	1.17	118.5
-0.0785	0.17	0.07	0.33	1	0	0	0.57	1	237.01
-0.0784	0.17	0.1	0.3	1	0.17	0	0.55	1.08	237.01
-0.0784	0.2	0.07	0.3	1	0.17	0	0.55	1.08	237.01
-0.0784	0.2	0.1	0.27	1	0.17	0	0.55	1.08	237.01
-0.0784	0.2	0.03	0.33	1	0	0	0.57	1	237.01
-0.0784	0.27	0.03	0.27	1	0.17	0	0.55	1.08	237.01
-0.0784	0.23	0	0.33	1	0	0	0.57	1	237.01
-0.0784	0.2	0.07	0.3	1	0.17	0	0.55	1.08	237.01
-0.0784	0.17	0.1	0.3	1	0.17	0	0.55	1.08	237.01
-0.0784	0.2	0.03	0.33	1	0	0	0.57	1	237.01
-0.0784	0.2	0.07	0.3	1	0	0	0.57	1	237.01
-0.0783	0.2	0.03	0.33	1	0.17	0	0.55	1.08	237.01
-0.0783	0.2	0.07	0.33	1	0	0	0.6	1	118.5
-0.0783	0.23	0.03	0.3	1	0	0	0.57	1	237.01
-0.0783	0.2	0.1	0.25	1	0	0	0.55	1	158.01
-0.0783	0.2	0.07	0.3	1	0	0	0.57	1	237.01

-0.0783	0.17	0.1	0.3	1	0.33	0	0.53	1.17	237.01
-0.0783	0.2	0.07	0.3	1	0.33	0	0.53	1.17	237.01
-0.0783	0.25	0.05	0.25	1	0	0	0.55	1	158.01
-0.0782	0.2	0.1	0.27	1	0.33	0	0.53	1.17	237.01
-0.0782	0.27	0	0.3	1	0	0	0.57	1	237.01
-0.0782	0.23	0	0.33	1	0	0	0.57	1	237.01
-0.0782	0.15	0.05	0.35	1	0	0	0.55	1	158.01
-0.0782	0.15	0.1	0.3	1	0.25	0	0.53	1.13	158.01
-0.0782	0.2	0.03	0.33	1	0	0	0.57	1	237.01
-0.0781	0.27	0.07	0.27	1	0.33	0	0.57	1.17	118.5
-0.0781	0.27	0.07	0.23	1	0	0	0.57	1	237.01
-0.0781	0.2	0.1	0.25	1	0.25	0	0.53	1.13	158.01
-0.0781	0.27	0.03	0.27	1	0	0	0.57	1	237.01
-0.0781	0.2	0.13	0.27	1	0.33	0	0.57	1.17	118.5
-0.0781	0.2	0.05	0.3	1	0.25	0	0.53	1.13	158.01
-0.0781	0.2	0.13	0.27	1	0.33	0	0.57	1.17	118.5
-0.0781	0.17	0.1	0.3	1	0.33	0	0.53	1.17	237.01
-0.0781	0.23	0.03	0.3	1	0.17	0	0.55	1.08	237.01
-0.0781	0.23	0	0.4	1	0	0	0.63	1	237.01
-0.078	0.13	0.1	0.33	1	0.17	0	0.55	1.08	237.01
-0.078	0.27	0.27	0.07	1	0.33	0	0.57	1.17	118.5
-0.078	0.23	0.03	0.37	1	0.17	0	0.62	1.08	237.01
-0.078	0.2	0	0.35	1	0	0	0.55	1	158.01
-0.078	0.2	0.07	0.3	1	0.17	0	0.55	1.08	237.01
-0.078	0.17	0.1	0.3	1	0.17	0	0.55	1.08	237.01
-0.078	0.2	0.2	0.2	1	0.33	0	0.57	1.17	118.5

-0.078	0.2	0.07	0.3	1	0.17	0	0.55	1.08	237.01
-0.078	0.17	0.1	0.3	1	0.17	0	0.55	1.08	237.01
-0.0779	0.2	0.05	0.3	1	0	0	0.55	1	158.01
-0.0779	0.2	0.03	0.33	1	0.17	0	0.55	1.08	237.01
-0.0779	0.2	0	0.37	1	0	0	0.57	1	237.01
-0.0779	0.17	0.03	0.37	1	0.17	0	0.55	1.08	237.01
-0.0779	0.17	0.23	0.17	1	0.5	0	0.52	1.25	237.01
-0.0779	0.17	0.07	0.33	1	0.17	0	0.55	1.08	237.01
-0.0779	0.2	0.03	0.33	1	0.17	0	0.55	1.08	237.01
-0.0779	0.2	0.1	0.25	1	0.25	0	0.53	1.13	158.01
-0.0779	0.27	0.07	0.2	1	0	0	0.53	1	118.5
-0.0779	0.33	0	0.27	1	0	0	0.6	1	118.5
-0.0778	0.27	0	0.27	1	0	0	0.53	1	118.5
-0.0778	0.27	0.07	0.27	1	0	0	0.6	1	118.5
-0.0778	0.2	0.05	0.3	1	0	0	0.55	1	158.01
-0.0778	0.2	0.1	0.25	1	0.25	0	0.53	1.13	158.01
-0.0778	0.25	0	0.3	1	0	0	0.55	1	158.01
-0.0778	0.2	0.13	0.2	1	0	0	0.53	1	118.5
-0.0778	0.27	0.07	0.23	1	0.17	0	0.55	1.08	237.01
-0.0778	0.2	0	0.43	1	0	0	0.63	1	237.01
-0.0778	0.13	0.2	0.2	1	0.33	0	0.5	1.17	118.5
-0.0777	0.27	0.13	0.27	1	0.33	0	0.63	1.17	118.5
-0.0777	0.27	0.13	0.27	1	0.33	0	0.63	1.17	118.5
-0.0777	0.27	0.07	0.23	1	0	0	0.57	1	237.01
-0.0777	0.2	0.03	0.37	1	0.17	0	0.58	1.08	237.01
-0.0777	0.2	0	0.4	1	0	0	0.6	1	118.5

-0.0777	0.4	0	0.2	1	0	0	0.6	1	118.5
-0.0777	0.17	0.07	0.33	1	0	0	0.57	1	237.01
-0.0777	0.2	0.03	0.4	1	0.17	0	0.62	1.08	237.01
-0.0777	0.2	0.07	0.33	1	0.33	0	0.57	1.17	118.5
-0.0776	0.23	0.03	0.3	1	0.17	0	0.55	1.08	237.01
-0.0776	0.27	0.13	0.27	1	0.33	0	0.63	1.17	118.5
-0.0776	0.27	0.13	0.2	1	0.33	0	0.57	1.17	118.5
-0.0776	0.33	0	0.27	1	0	0	0.6	1	118.5
-0.0775	0.27	0.13	0.2	1	0.33	0	0.57	1.17	118.5
-0.0775	0.47	0	0.13	1	0	0	0.6	1	118.5
-0.0775	0.27	0.2	0.2	1	0.33	0	0.63	1.17	118.5
-0.0775	0.13	0.2	0.27	1	0.33	0	0.57	1.17	118.5
-0.0775	0.27	0.13	0.2	1	0.33	0	0.57	1.17	118.5
-0.0774	0.27	0.07	0.33	1	0.33	0	0.63	1.17	118.5
-0.0774	0.2	0	0.45	1	0	0	0.65	1	158.01
-0.0774	0.2	0.03	0.4	1	0.17	0	0.62	1.08	237.01
-0.0774	0.37	0	0.3	1	0	0	0.67	1	237.01
-0.0774	0.27	0.27	0.13	1	0.33	0	0.63	1.17	118.5
-0.0773	0.27	0.13	0.27	1	0.33	0	0.63	1.17	118.5

3.3 Pt(643)

Data used to generate Figure 6 in the manuscript.

Table S3: Pt(643) LEME structures data: Free energy per unit area (G/A), Coverage of CO on the terrace top site (θ_t(T)), bridge site (θ_t(B)), hollow site (θ_t(H)) and on the step edge top site (θ_e(T)), bridge site (θ_e(B)), hollow site (θ_e(H)), total coverage of CO on the terrace (θ_t) and on the step edge (θ_e) and the surface area of the unit cell (A)

G/A (eV/Å²)	θ_t(T)	θ_t(B)	θ_t(H)	θ_e(T)	θ_e(B)	θ_e(H)	θ_t	θ_e
-0.0845	0.29	0.00	0.14	1.00	0.00	0.00	0.43	1.00
-0.0843	0.14	0.07	0.14	1.00	0.00	0.00	0.36	1.00
-0.0839	0.21	0.00	0.14	1.00	0.00	0.00	0.36	1.00
-0.0838	0.14	0.07	0.14	1.00	0.00	0.00	0.36	1.00
-0.0837	0.14	0.07	0.07	1.00	0.00	0.00	0.29	1.00
-0.0836	0.00	0.07	0.29	1.00	0.00	0.00	0.36	1.00
-0.0835	0.14	0.00	0.21	1.00	0.00	0.00	0.36	1.00
-0.0835	0.14	0.00	0.21	1.00	0.00	0.00	0.36	1.00
-0.0834	0.14	0.07	0.14	1.00	0.00	0.00	0.36	1.00
-0.0834	0.14	0.00	0.29	0.83	0.00	0.00	0.43	0.83
-0.0831	0.21	0.00	0.21	0.83	0.00	0.00	0.43	0.83
-0.0831	0.29	0.00	0.07	1.00	0.00	0.00	0.36	1.00
-0.0830	0.00	0.00	0.36	1.00	0.00	0.00	0.36	1.00
-0.0829	0.14	0.21	0.07	1.00	0.00	0.00	0.43	1.00
-0.0829	0.07	0.07	0.21	1.00	0.00	0.00	0.36	1.00
-0.0826	0.29	0.07	0.14	0.83	0.00	0.00	0.50	0.83
-0.0825	0.21	0.00	0.14	1.00	0.00	0.00	0.36	1.00
-0.0824	0.14	0.07	0.07	0.83	0.17	0.00	0.29	1.00
-0.0821	0.21	0.07	0.14	0.83	0.17	0.00	0.43	1.00
-0.0819	0.36	0.21	0.00	0.83	0.00	0.00	0.57	0.83

4 CO-Surface vs CO-CO lateral interaction

4.1 CO-Surface Interaction

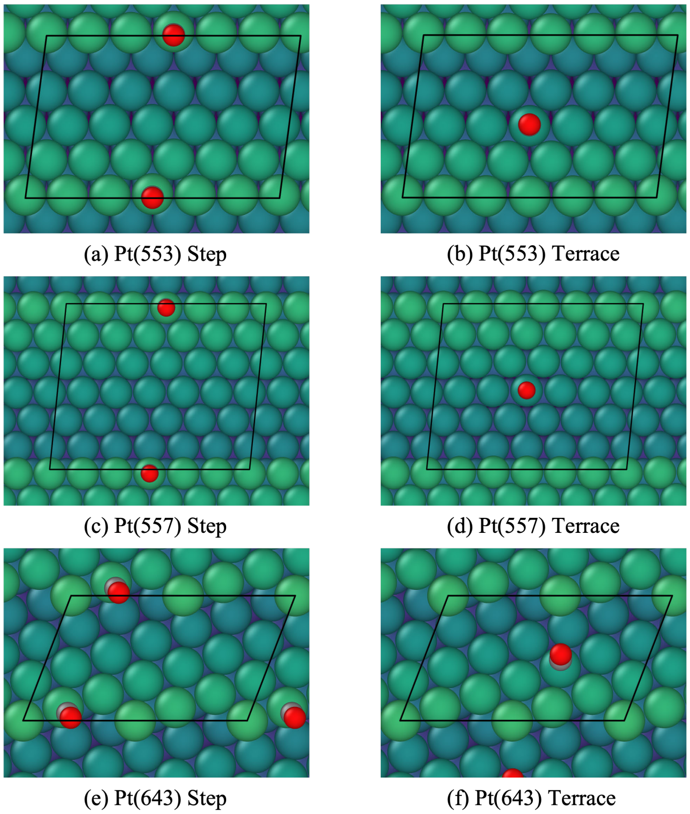

Figure S6: Configurations used to compare adsorption energy of CO on step edge and the terrace.

Table S4: Comparison the adsorption energy of CO on the step edge and the terrace.

	Step (eV)	Terrace (eV)
Pt(553)	-1.84	-1.21
Pt(557)	-1.85	-1.33
Pt(643)	-1.81	-1.25

5 Neural Network Evaluation

5.1 Pt(553)

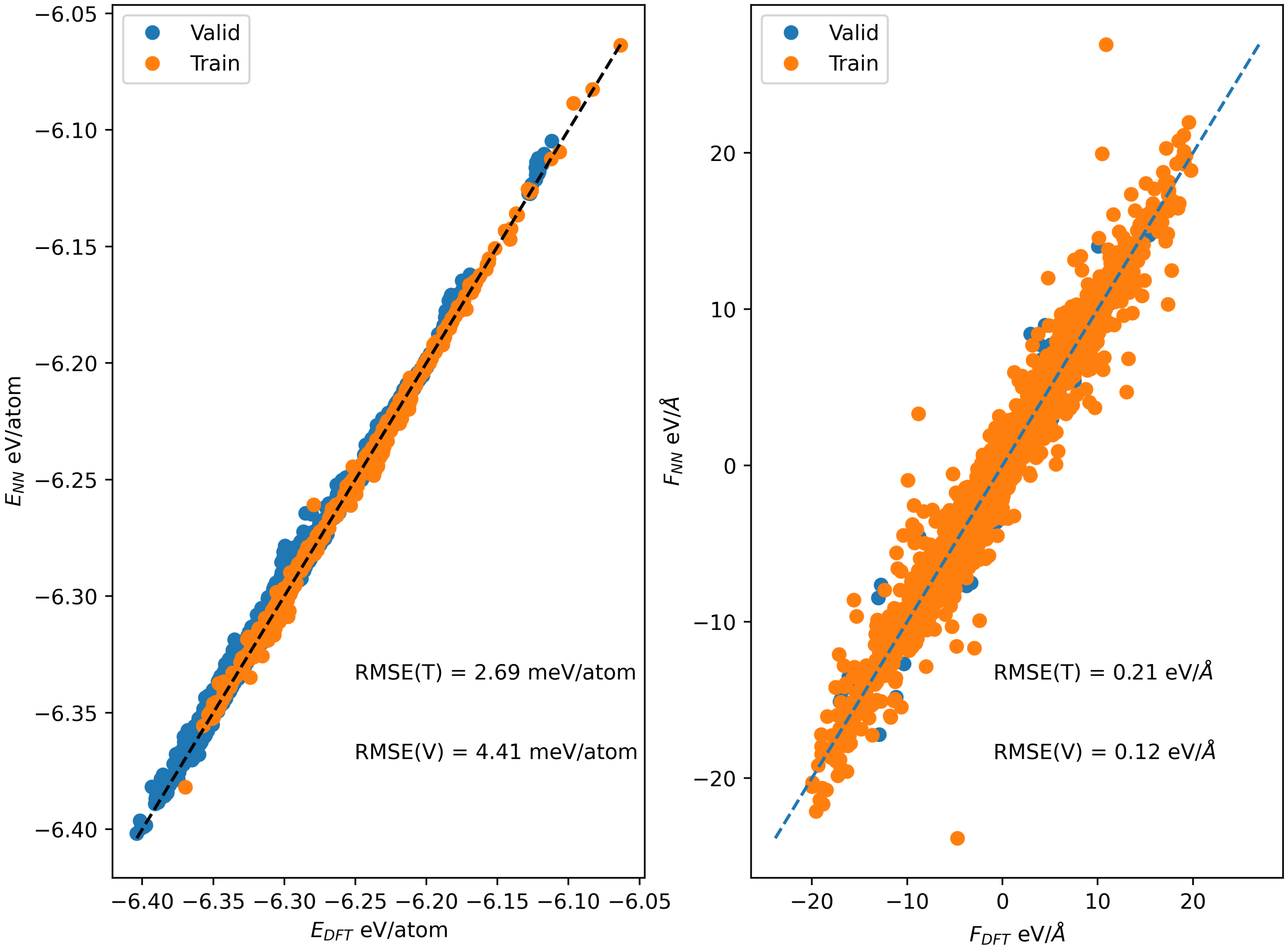

Figure S7: Parity plot comparing the reference DFT energies and forces with the neural network estimates for Pt(553)

5.2 Pt(557)

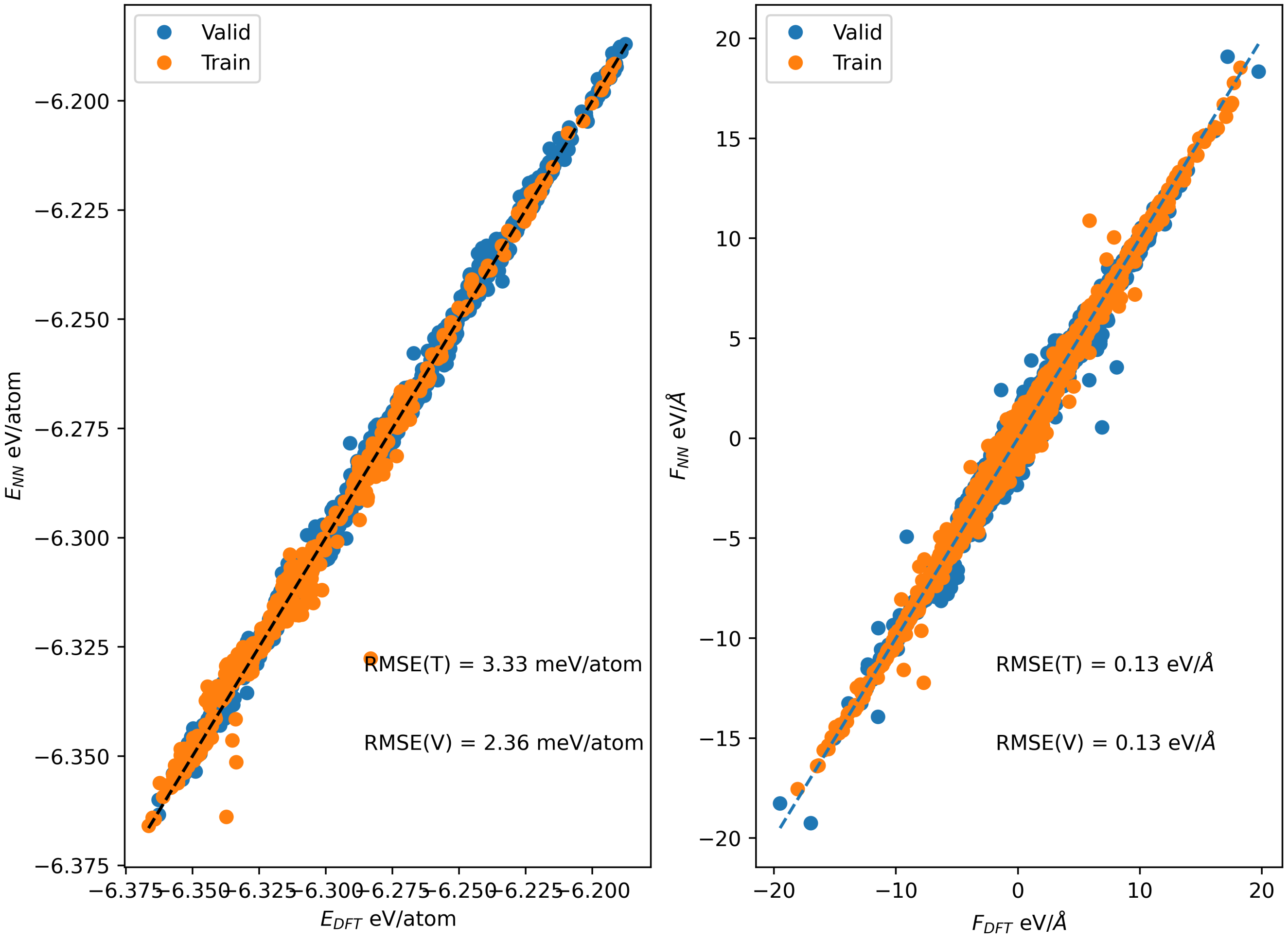

Figure S8: Parity plot comparing the reference DFT energies and forces with the neural network estimates for Pt(557)

5.3 Pt(643)

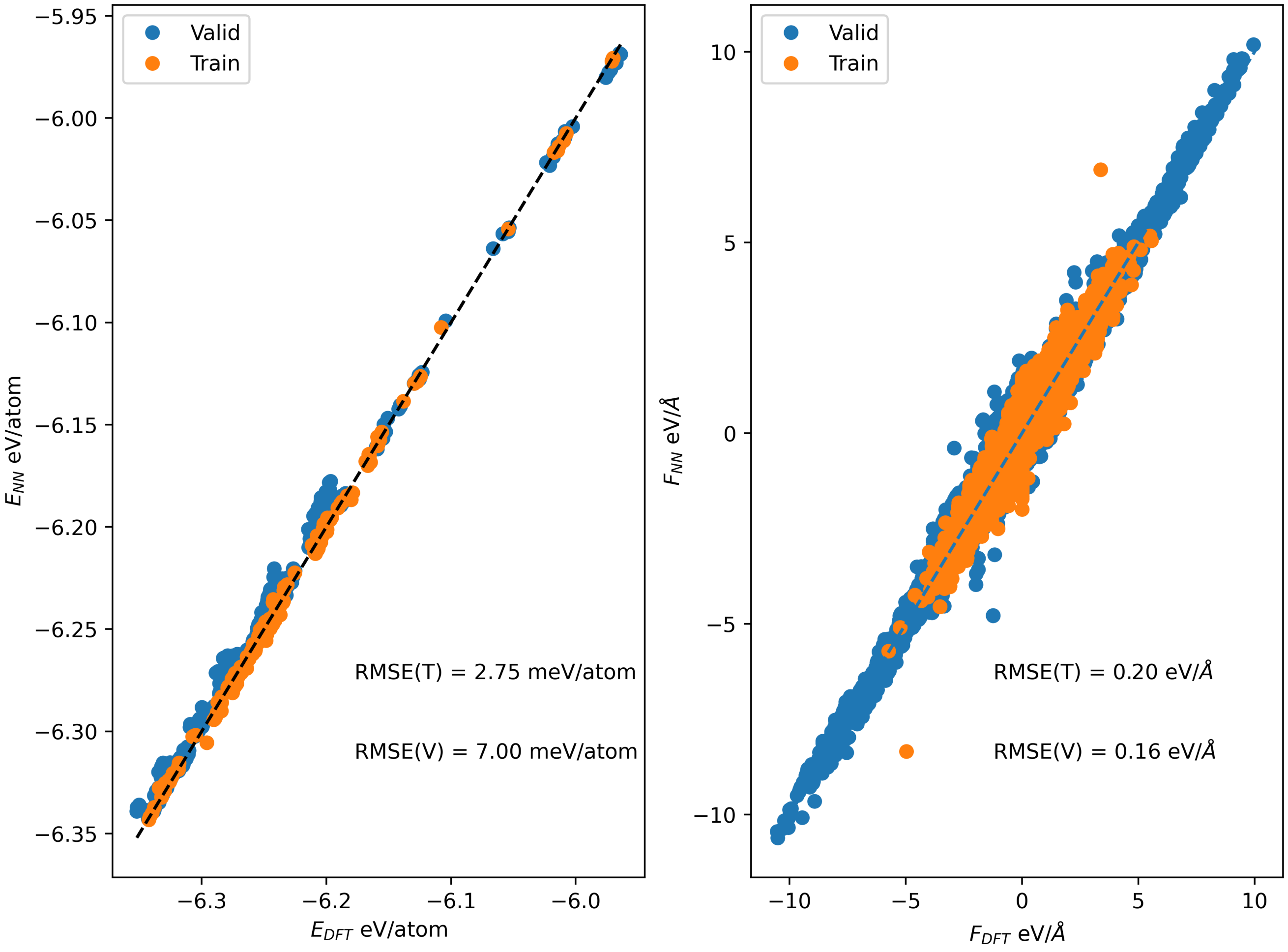

Figure S9: Parity plot comparing the reference DFT energies and forces with the neural network estimates for Pt(643)

5.4 Pt(111)

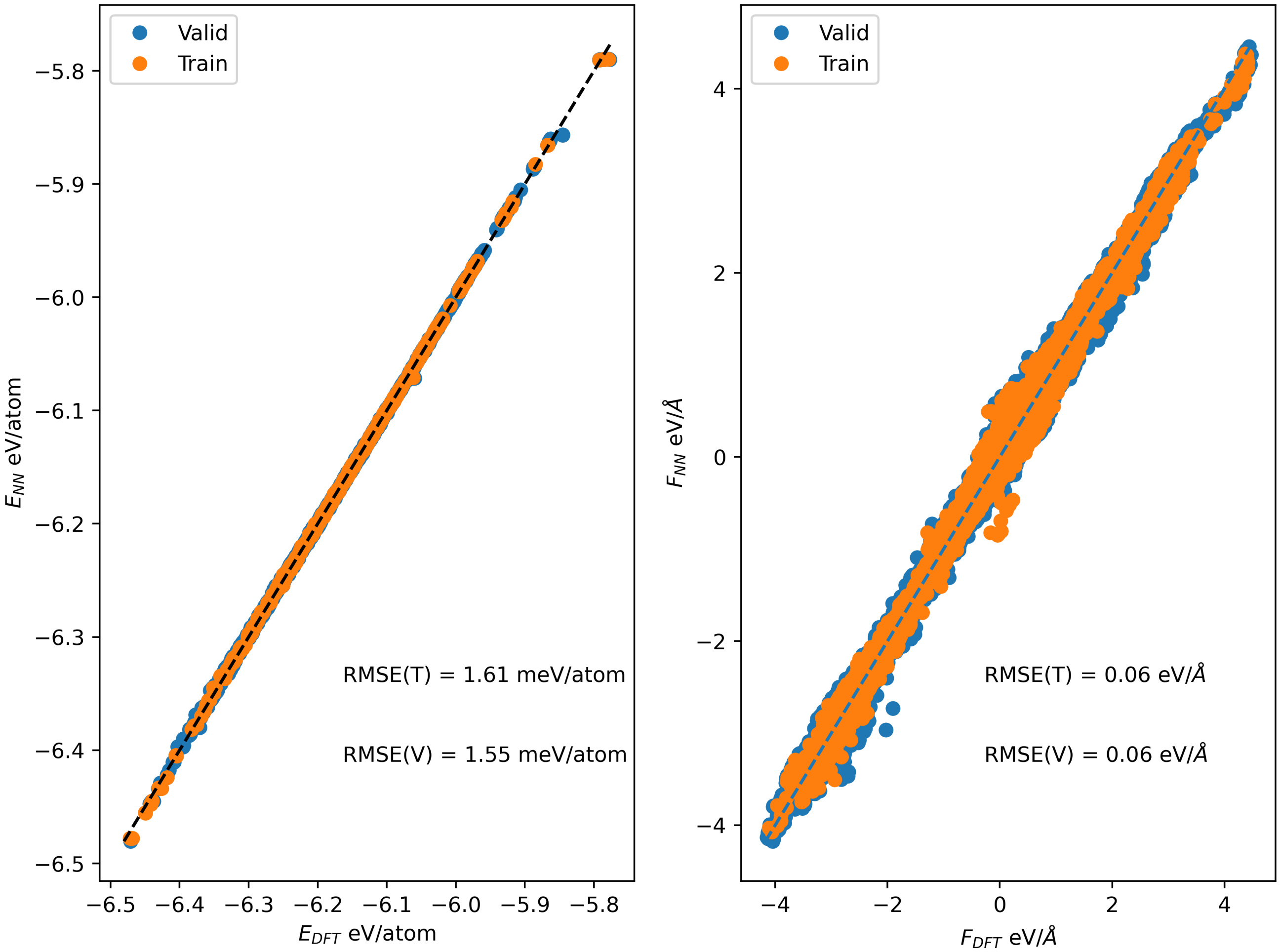

Figure S10: Parity plot comparing the reference DFT energies and forces with the neural network estimates for Pt(111)

5.5 Low Coordination adsorption sites

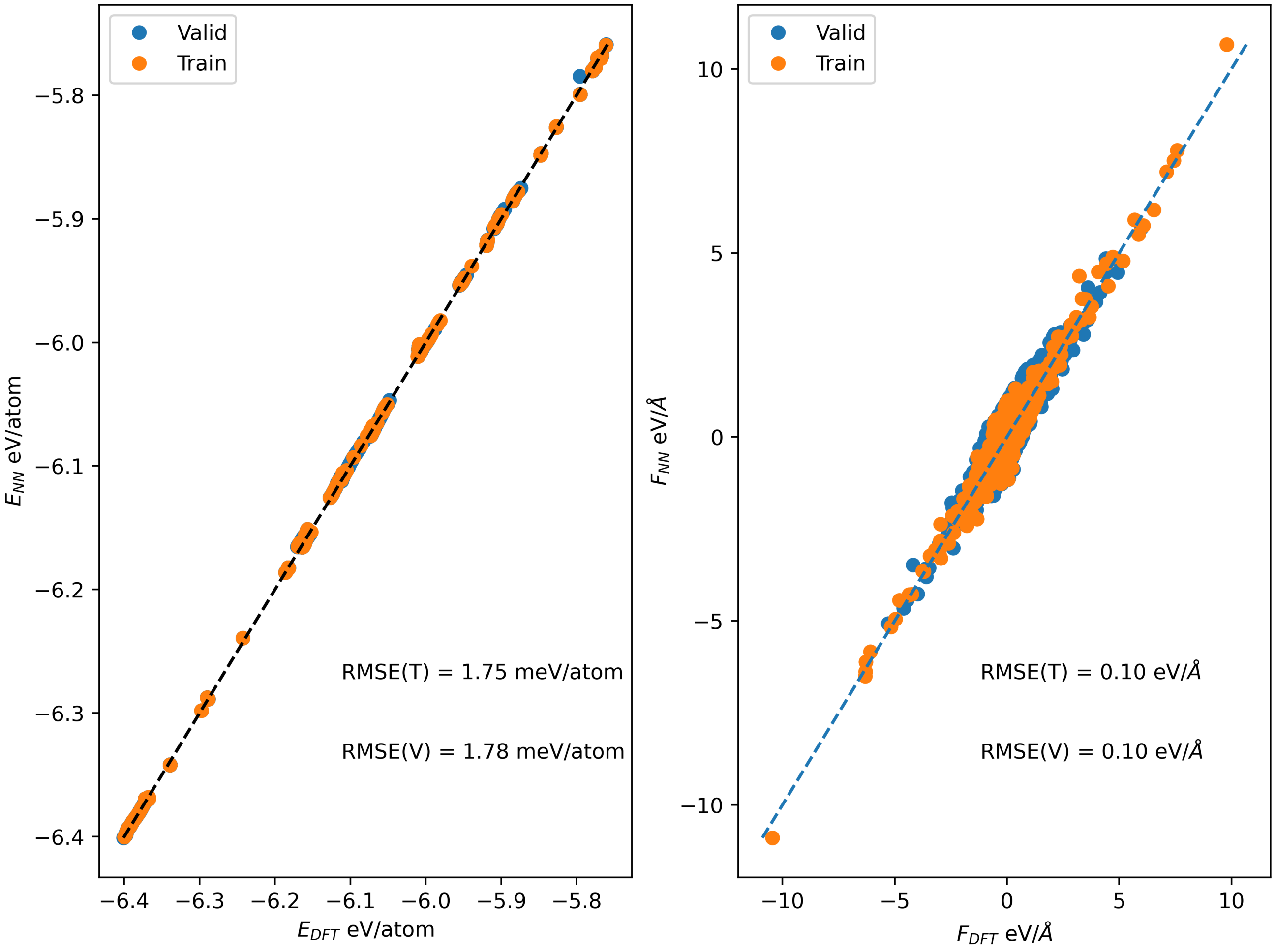

Figure S11: Parity plot comparing the reference DFT energies and forces with the neural network estimates for structures with low coordination adsorption sites
